# Nuclear translocation of FGFR1 and FGF2 in pancreatic stellate cells facilitates pancreatic cancer cell invasion

**DOI:** 10.1002/emmm.201302698

**Published:** 2014-02-06

**Authors:** Stacey J Coleman, Athina-Myrto Chioni, Mohammed Ghallab, Rhys K Anderson, Nicholas R Lemoine, Hemant M Kocher, Richard P Grose

**Affiliations:** 1 Centre for Tumour Biology, Barts Cancer Institute – a CRUK Centre of Excellence, Queen Mary University of LondonLondon, UK; 2 Centre for Molecular Oncology, Barts Cancer Institute – a CRUK Centre of Excellence, Queen Mary University of LondonLondon, UK; 3 Barts and the London HPB Centre, The Royal London Hospital, Barts Health NHS TrustLondon, UK

**Keywords:** FGF signalling, human cancer, invasion, organotypic cultures

## Abstract

Pancreatic cancer is characterised by desmoplasia, driven by activated pancreatic stellate cells (PSCs). Over-expression of FGFs and their receptors is a feature of pancreatic cancer and correlates with poor prognosis, but whether their expression impacts on PSCs is unclear. At the invasive front of human pancreatic cancer, FGF2 and FGFR1 localise to the nucleus in activated PSCs but not cancer cells. *In vitro*, inhibiting FGFR1 and FGF2 in PSCs, using RNAi or chemical inhibition, resulted in significantly reduced cell proliferation, which was not seen in cancer cells. In physiomimetic organotypic co-cultures, FGFR inhibition prevented PSC as well as cancer cell invasion. FGFR inhibition resulted in cytoplasmic localisation of FGFR1 and FGF2, in contrast to vehicle-treated conditions where PSCs with nuclear FGFR1 and FGF2 led cancer cells to invade the underlying extra-cellular matrix. Strikingly, abrogation of nuclear FGFR1 and FGF2 in PSCs abolished cancer cell invasion. These findings suggest a novel therapeutic approach, where preventing nuclear FGF/FGFR mediated proliferation and invasion in PSCs leads to disruption of the tumour microenvironment, preventing pancreatic cancer cell invasion.

## Introduction

Pancreatic cancer (pancreatic ductal adenocarcinoma, PDAC) carries a dismal prognosis (Coupland *et al*, 2012). The dense desmoplastic stroma, precluding conventional chemotherapeutic drugs and radiotherapy from accessing the cancer cells, may be one of the reasons for its poor prognosis (Olive *et al*, 2009). Improved understanding of the role of the stroma in pancreatic cancer is now possible due to the isolation, and *in vitro* culture, of pancreatic stellate cells (PSCs), the main cells responsible for desmoplasia in PDAC (Apte *et al*, 1998; Erkan *et al*, 2012; Kadaba *et al*, 2013).

Activation of PSCs in PDAC results in their differentiation into pancreatic myo-fibroblasts, as identified by cell markers such as α-smooth muscle actin (αSMA) and vimentin (Apte *et al*, 1999). A number of *in vitro* and *in vivo* studies have shown that cross-talk between PSCs and PDAC cells facilitates local tumour growth as well as regional and distant metastatic spread of PDAC (Apte *et al*, 1999, 2004; Bachem *et al*, 2005; Hwang *et al*, 2008; Vonlaufen *et al*, 2008; Xu *et al*, 2010). Furthermore, by rendering PSCs quiescent, for example by restoring physiological retinol stores, we have demonstrated that the homeostatic cross-talk, hijacked by cancer, can be re-instated, to reduce pancreatic cancer cell survival and invasion significantly both *in vitro* and *in vivo* (Froeling *et al*, 2011). Multiple signalling cascades are altered by retinol treatment of stellate cells; one such being the fibroblast growth factor (FGF) signalling cascade, known already to play a critical role in pancreatic organogenesis (Kim & Hebrok, 2001).

FGFs are heparin binding polypeptides, most of which are secreted ligands that signal through four high affinity transmembrane FGF receptors (FGFRs) (Ornitz & Itoh, 2001). As well as playing a vital role in developmental processes, expression of FGFs and their receptors has also been implicated in a number of cancers including breast, prostate, brain, lung and endometrial tumours (Turner & Grose, 2010). FGF2, in particular, has the ability to stimulate the growth of fibroblasts, endothelial and epithelial cells. Classically, FGF2 mediates its biological effects by binding to FGFRs, leading to activation of a number of signalling cascades; predominately the MAPK/MEK/ERK pathway (Corson *et al*, 2003). Multiple forms of FGF2 exist. Both high-and low-molecular weight (HMW and LMW, respectively) forms of FGF2 can localise directly to the nucleus. Nuclear FGF2 can promote proliferation, growth, differentiation and functional activation in a variety of cell types (Moffett *et al*, 1996; Clarke *et al*, 2001; Dunham-Ems *et al*, 2009). Furthermore, reactive astrocytes show accumulation of nuclear FGF2, whereas FGF2 is predominately cytoplasmic in quiescent cells (Clarke *et al*, 2001), suggesting that these biological effects are a result of interaction of FGF2 with nuclear targets.

FGFRs can also be targeted to the nucleus. In particular, full length FGFR1 and HMW FGF2 have been shown to co-localise in the nuclear matrix, where together they may co-activate transcription and thus control proliferation (Stachowiak *et al*, 1996a, 1997b, 2003; Reilly & Maher, 2001; Peng *et al*, 2002; Dunham-Ems *et al*, 2009). For example, in human glial cells, the accumulation of FGF2 and FGFR1 in the nucleus is associated with mitotic activation and hypertrophy (Stachowiak *et al*, 1996a, 1997a,b1997b; Joy *et al*, 1997; Peng *et al*, 2001). Thus, nuclear FGFR1 may mediate the effects of nuclear FGF2. Recently, we demonstrated that FGFR1 cleavage and nuclear translocation results in upregulation of an invasive gene signature in breast cancer (Chioni & Grose, 2012).

In PDAC, a number of FGFs and FGFRs are over-expressed, correlating with poor patient outcome (Yamanaka *et al*, 1993; Leung *et al*, 1994a,b; Kornmann *et al*, 1997, 1998, 2002; Yamazaki *et al*, 1997; Ishiwata *et al*, 1998). For example, over-expression of the IIIC isoform of FGFR1 in pancreatic cancer can promote tumourigenesis (Kornmann *et al*, 2001, 2002; Chen *et al*, 2010; Tian *et al*, 2012). Initial experiments exploring FGF2 expression in PDAC described FGF2 apparent in the nuclei of many cancer cells but not in normal pancreatic tissue, suggesting intranuclear FGF2 may be important in this cancer (Yamanaka *et al*, 1993; Leung *et al*, 1994). However, the function of nuclear FGF2 in PDAC, particularly with respect to PSCs, has not been explored.

Here we show for the first time that, within PSCs, nuclear translocation of FGFR1, along with FGF2, is necessary for maintaining PSC proliferation. This nuclear localisation is critical in mediating the invasion of PSCs and, consequently, cancer cells. Our data provide a rationale for further targeting of this novel stromal pathway in clinical trials, in conjunction with conventional chemotherapy.

## Results

### *In vivo* nuclear FGFR1 and FGF2 in human PDAC

**Figure 1:**
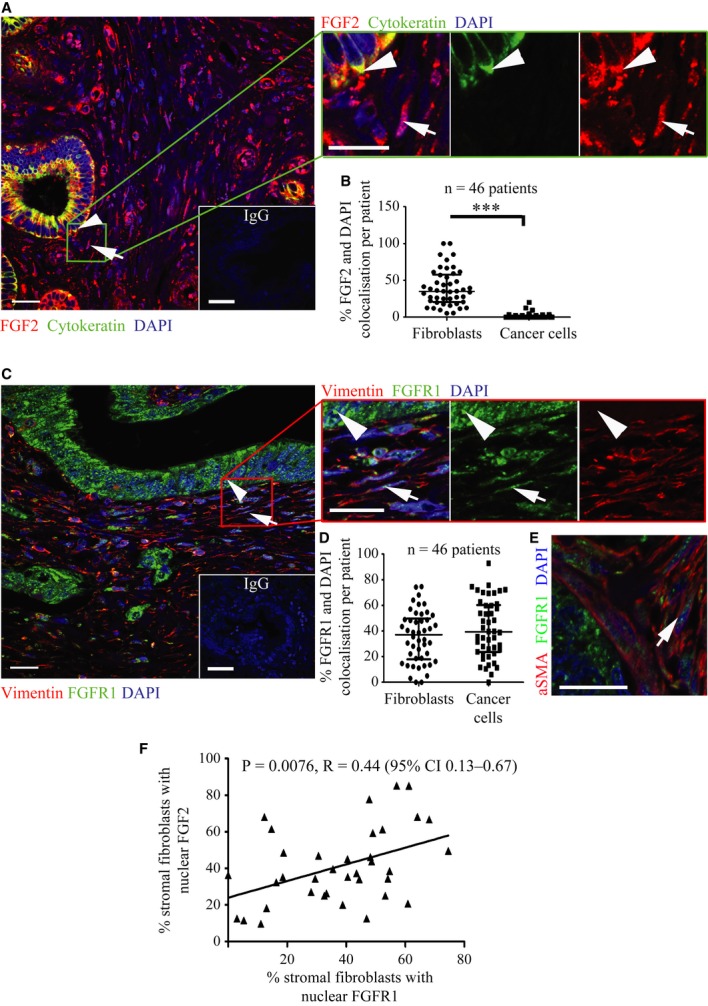
FGF2 and FGFR1 localise to the nucleus of fibroblasts in human PDAC tissues.
Pancreatic cancer tissue showed cytokeratin positive (green, arrowhead) epithelial tumour cells with cytoplasmic FGF2 (red); however, cytokeratin negative stromal cells with fibroblastic morphology (arrow) showed nuclear FGF2 staining (red), demonstrated clearly in the side panel. DAPI stains the nuclei. Inset box shows IgG control.FGF2 and DAPI pixel co-localisation analysis (of 46 patients, at least one TMA core analysed per patient) performed by confocal microscopy (See Supplementary Fig 1 and Methods) confirmed the presence of nuclear FGF2 in 35% of stromal cells but not in tumour cells. ***P < 0.0001, Mann–Whitney *U*-test (B, D). Data summary represented by median ± interquartile range.Similarly, FGFR1 (green) was present in the nuclei of fibroblasts, as identified by vimentin expression (red, arrow). Vimentin negative cells with epithelial, glandular morphology showed cytoplasmic and nuclear FGFR1 (arrow head) as shown in side panel consistent with A. Inset box shows IgG control.FGFR1 and DAPI pixel co-localisation analysis performed (of 46 patients, at least one TMA core analysed per patient) by confocal microscopy, as above, confirmed presence of nuclear FGFR1 in 37% of stromal cells and 39% of cancer cells. Mann–Whitney *U*-test (B, D). Data summary represented by median ± interquartile range.Results in C and D were confirmed by independent co-staining of serial sections with αSMA (red) and FGFR1 (green).Significant correlation was found between the presence of FGF2 and FGFR1 in the nuclei of stromal fibroblasts, from the 36 patients in B and D who had been scored for both FGFR1 and FGF2. Data information: Scale Bar: 20 μm, IgG 100 μm.

Cell-specific expression of FGF2 and FGFR1 in human PDAC was assessed by double staining (FGF2/cytokeratin, FGFR1/vimentin or FGFR1/αSMA) PDAC tissue microarrays (Fig 1). FGF2 was expressed universally in PDAC tissue. In contrast to the cytoplasmic expression of FGF2 in cancer cells, many (∼35%) myo-fibroblasts (activated PSCs (Apte *et al*, 1999)) expressed nuclear FGF2, as evident from co-localisation analysis (Supplementary Fig 1, Fig 1B). There was also nuclear and cytoplasmic staining of FGFR1 in approximately 39% of cancer cells and approximately 37% of myo-fibroblasts (Fig 1C–E). Furthermore, in patient samples, there was positive correlation of nuclear FGF2 and FGFR1 in myo-fibroblasts, but not in cancer cells (Fig 1F, Supplementary Fig 1B).

### *In vitro* FGFR1 and FGF2 expression in human pancreatic cancer and stellate cells

**Figure 2:**
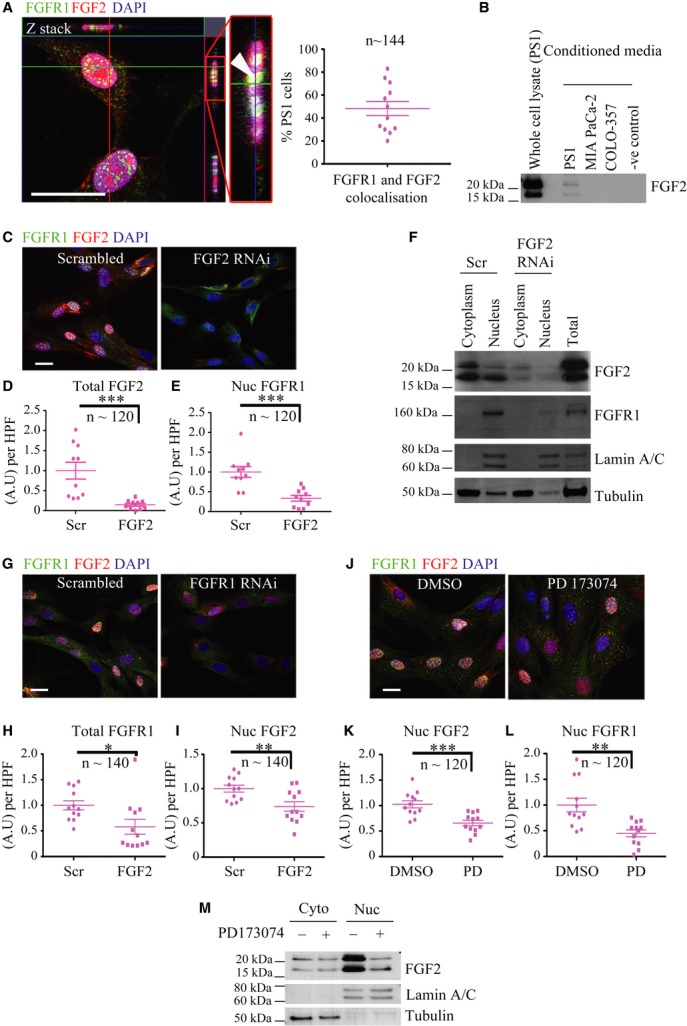
Pancreatic stellate cells show co-dependency of nuclear FGFR1 and FGF2 localisation. A Pancreatic stellate cells (PS1 cell line) showed punctate, nuclear speckles of FGFR1 staining (green) and diffuse nuclear FGF2 (red), with co-localisation (yellow, indicated by arrow head) confirmed by optical sectioning through the Z-axis (Z-stack) and pixel co-localisation techniques (50% of PS1 cells). Nuclear FGF2 was apparent only in those cells with nuclear FGFR1. B Serum-free conditioned media confirmed PS1, but not cancer cells, secrete high-and low-molecular weight (HMW 24 kDa and LMW 18 kDa, respectively) forms of FGF2. Whole cell lysate (untreated PS1 cells) and serum free medium were used as positive and negative controls respectively. C–E RNAi mediated knock-down of FGF2 (FGF2) resulted in a significant reduction in nuclear FGFR1 in PS1 cells in comparison to scrambled RNAi (Scr), as demonstrated by microscopic analysis. (D) ***P = 0.0009, (E) ***P = 0.0004. Student's *t*-test. Data summary represented by mean ± s.e.m. F Sub-cellular fractionation and subsequent immuno-blotting confirmed that FGFR1 expression was dependent upon FGF2. Total lysate was used as a positive control. Lamin A/C and tubulin were used as markers of fraction purity and loading control. G–I FGFR1 RNAi (FGFR) resulted in significant reduction in nuclear FGF2 compared to scrambled RNAi (Scr) treated PS1 cells. (H) *P = 0.0220. (I) **P = 0.0062. Student's *t*-test. Data summary represented by mean ± s.e.m. J–L FGFR inhibitor treatment (PD173074, 2 μM, 48 h) resulted in significant reduction in nuclear FGF2 and FGFR1 as compared to vehicle control (DMSO). (K) ***P = 0.0004, (L) **P = 0.0013. Student's *t*-test. Data summary represented by mean ± s.e.m. M Reduction in nuclear HMW and LMW isoforms of FGF2 upon FGFR inhibition (PD173074) was confirmed by sub-cellular fractionation. Data information: Scale Bar, 20 μm. For analysis of nuclear FGFR1 and FGF2, each data point shown represents an average of total or nuclear FGFR1 or FGF2 per field per experiment. Several fields were counted per experiment. The total number of PS1 cells analysed is recorded in the figure (n). For all data, images are representative of three independent experiments. Source data are available for this figure.

The vast majority of αSMA positive fibroblasts in pancreatic cancer represent activated pancreatic stellate cells (Vonlaufen *et al*, 2008; Froeling *et al*, 2011). With this in mind, we examined the expression and localisation of FGFR1 and FGF2 *in vitro*, using immunofluorescence and Western blot analysis to screen a panel of poorly-and well-differentiated PDAC cell lines, normal pancreatic ductal epithelial cell lines (HPDE (Furukawa *et al*, 1996) and DEC-hTERT (Li *et al*, 2009)) and immortalised [PS1(Froeling *et al*, 2011)] and primary stellate cells (Supplementary Figs 2 and 3). MCF7 (breast cancer) cells were used as a positive control for FGFR1 expression (Chioni & Grose, 2012). The low level of plasma membrane staining is a result of the technique used to permeabilise the cells, and cytoplasmic localisation of FGFR1 has been described previously (Maher, 1996). Whilst FGFR1 expression was weak in normal epithelial cell lines, it was stronger in many of the poorly differentiated cancer cells lines (Supplementary Fig 2E). In contrast to the cytoplasmic localisation in well-differentiated cancer cell lines, FGFR1 was mainly nuclear in stellate cells (speckled distribution) and some poorly-differentiated cancer cell lines (Fig 2A, Supplementary Figs 2 and 3B).

Stellate cells showed strong expression and secretion of both HMW and LMW FGF2 isoforms (2Fig B), with confocal microscopy analysis revealing that FGF2 was localised predominantly within the nuclei (nucleolar and diffuse nuclear staining, Fig 2A). Furthermore, FGF2 was apparent only in stellate cells in which FGFR1 was nuclear (Fig 2A, Supplementary Fig 2D and 3B). In contrast, most cancer cell lines expressed moderate levels of both HMW and LMW FGF2 isoforms, which appeared cytoplasmic or peri-nuclear. Several well-differentiated lines showed little or no expression of FGF2 (Supplementary Fig 2B and E).

### Nuclear FGFR1 and FGF2 in stellate cells

In order to assess the relationship between nuclear FGF2 and FGFR1 in stellate and cancer cells, PS1, MIA PaCa-2 and COLO-357 cell lines were subjected to RNAi mediated knock-down of FGF2. Efficient FGF2 knock-down had no effect on nuclear FGFR1 in PDAC cells (Supplementary Fig 4A and B, 5A). In contrast, PS1 cells demonstrated a significant reduction in nuclear FGFR1, upon FGF2 knock-down (Fig 2C–E).

Further analyses, by sub-cellular fractionation, confirmed that FGF2 (HMW and LMW) and FGFR1 localised to the nucleus in PS1 cells, and that nuclear localisation of FGFR1 was dependent on nuclear FGF2 (Fig 2F). Similarly, knock-down of FGFR1 in stellate cells resulted in a significant reduction of nuclear FGF2 (Fig 2G–I, Supplementary Fig 4C). In contrast to PS1 cells, no effect was seen on nuclear FGFR1 following FGF2 knock-down in PDAC cells (Supplementary Fig 5A).

In order to test whether nuclear FGFR1 and FGF2 were dependent upon FGFR signalling, we treated cells with a well-validated chemical inhibitor of FGFR, PD173074. PD173074, in contrast to many other receptor tyrosine kinase inhibitors, is highly specific to FGFRs (Mohammadi *et al*, 1998a). Preliminary experiments determined the correct dosing schedule and clearly demonstrated a dose-dependent effect (Supplementary Fig 5B and C). Treatment with PD173074 at 2 μM resulted in a significant reduction in nuclear FGF2 (both LMW and HMW) and FGFR1 in PS1 cells (Fig 2J–M). However, no effect was seen in COLO-357 cells (Supplementary Fig 5D and E).

Western blotting of PS1 cells, stimulated with FGF2, confirmed that PD173074 blocked activation of the fibroblast growth factor receptor substrate 2 (FRS2) -extracellular signal-regulated kinase (ERK) pathway (Supplementary Fig 6A). Interestingly, abolishing FRS2 using RNAi showed a significant effect on both nuclear FGFR1 and FGF2, confirming that receptor activation is important for nuclear translocation of both receptor and ligand (Supplementary Fig 6B–F). Therefore, to assess whether exogenous FGF2 was able to induce nuclear accumulation of FGFR1 and FGF2, we treated serum starved PS1 cells with recombinant FGF2 for 2 h. FGF2 stimulation led to significantly increased nuclear FGFR1 and FGF2 within 15 min of treatment (Supplementary Fig 6G–I).

### Nuclear FGFR1 and FGF2 regulate proliferation of stellate cells

**Figure 3:**
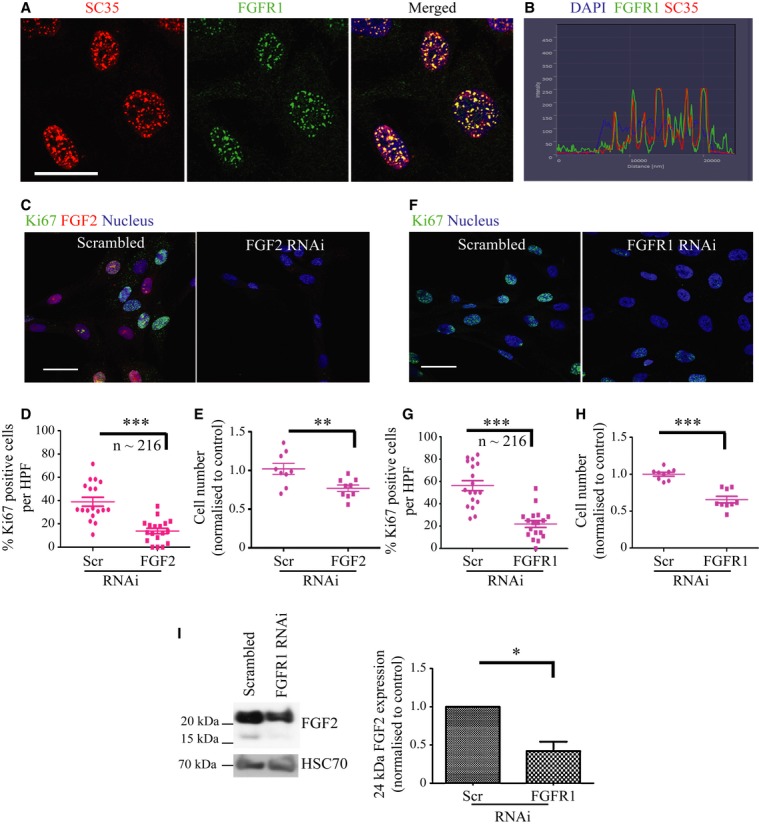
Stellate cell nuclear FGFR1 is associated with PSC proliferation. A FGFR1 (green) co-localised with splicing assembly factor, SC35 (red) at distinct nuclear speckles within the nuclei of stellate cells. B Pixel intensity analysis confirmed this co-localisation, with perfect overlap of red and green staining signals within the nucleus (blue). C–H RNAi-mediated knock-down of FGF2 (C, D) and FGFR1 (F, G) in stellate cells resulted in a significant reduction in proliferative index (% Ki67 positive cells, D, G; each data point represents percent of cells positive for Ki67 per field, multiple fields were taken per experiment. A total of 216 PS1 cells were analysed.) and, consequently, total cell count (E, H; each data point refers to one technical repeat. Three technical repeats were carried out per experiment, each experiment was carried out in triplicate.), relative to scrambled RNAi control (Scr). D, ***P = 0.0001. E, **P = 0.0081. G, ***P=<0.0001. H, ***P = <0.0001. Student's *t*-test. Data summary is represented by mean ± s.e.m. I RNAi-mediated knock-down of FGFR1 in stellate cells resulted in a significant reduction in FGF2 expression (HMW form). *P = 0.0423. Student's *t*-test. Data summary is represented by mean ± s.e.m. Data information: Scale Bar: 20 μm. For all data, images are representative of three independent experiments. Source data are available for this figure.

Stellate cells demonstrated nuclear FGFR1 speckles, a pattern demonstrated previously as domains for RNA Pol II-mediated transcription as well as co-transcriptional, pre-mRNA processing (Stachowiak *et al*, 1996b; Somanathan *et al*, 2003). We confirmed the FGFR1 localisation at these sites by co-staining with a validated marker, spliceosome assembly factor SC-35 (Crispino *et al*, 1994) (Fig 3A and B).

FGF2 and FGFR1 knock-down resulted in a significant reduction in total cell count for PS1, further substantiated by a reduction in the proliferative index (% Ki67 positive cells) (Fig 3C–H). In agreement with their role in stellate cell proliferation, nuclear FGF2 and FGFR1 were associated with Ki67 positivity in PS1 cells (Supplementary Fig 7A–C), however no effect was seen in cancer cells upon FGF2 or FGFR1 knockdown (Supplementary Fig 4C and 8A–F). Moreover, knock-down of FGFR1 resulted in a significant reduction of FGF2 (HMW) in PSCs, but not in cancer cells (Fig 3I, Supplementary Fig 8J).

**Figure 4:**
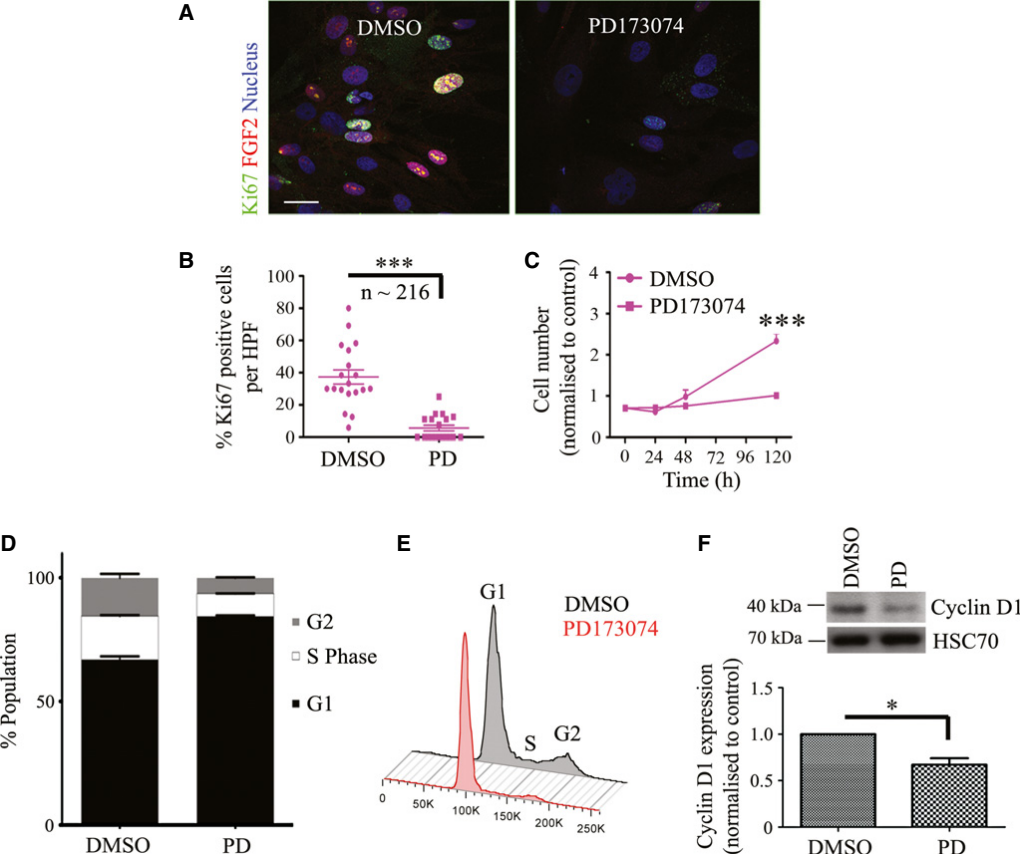
Blocking FGFR signalling results in a G1 block in PSCs. A–C Drug-mediated inhibition of FGFR (PD173074, 2 μM) resulted in a significant reduction in proliferative index (Ki67 positive cells, A, B; each data point represents percent of cells positive for Ki67 per field, multiple fields were taken per experiment. A total of 216 cells stellate cells were analysed.) and cell growth (C; each data point refers to one technical repeat. Three technical repeats were carried out per experiment.) after 5 days treatment compared to vehicle (DMSO) treated cells. B, ***P = <0.0001. C, ***P = <0.0001 (120 h). Student's *t*-test. Data summary is represented by mean ± s.e.m. D,E Cell cycle analysis after treatment with PD170374 (PD) for 48 h revealed a G1 cell cycle block in stellate cells compared to vehicle-treated (DMSO) cells. Representative cell cycle data after propidium iodide staining and analysis by FACS, are shown. F PD170374 treatment (PD) resulted in significant reduction in Cyclin D1 expression. HSC70 was used as a loading control. *P = 0.0414. Student's *t*-test. Data summary is represented by mean ± s.e.m. Data information: Scale Bar: 20 μm. For all data, images are representative of three independent experiments, except cell cycle data, which are representative of two independent experiments. Source data are available for this figure.

Furthermore, PD173074 treatment did not affect cancer cell proliferation but caused a marked reduction in PS1 cell number and proliferative index (Fig 4A–C, Supplementary Fig 8G–I), which was associated with a G1 cell-cycle arrest (Fig 4D and E). Concurrent with these observations, we could demonstrate a significant reduction in expression of the G1 phase cyclin, cyclin D1, in PS1 cells, but not cancer cells, upon FGFR inhibition (Fig 4F, Supplementary Fig 8K).

**Figure 5:**
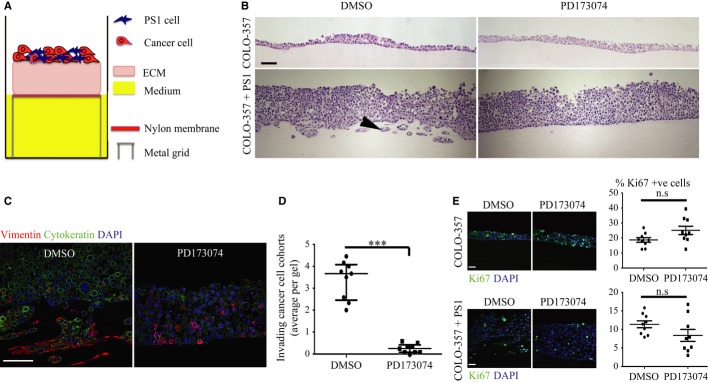
FGFR inhibition in stellate cells leads to reduced cancer cell invasion.
Schematic model of raised air-liquid organotypic culture model as described in Methods.A 2 × 2 experimental design, with COLO-357 cells alone or PS1 and COLO-357 cells co-cultured in the presence or absence of PD173074 (2 μM) for 14 days, was used to detect consequences of inhibition of FGFR1 signalling. H&E images showed that COLO-357 cells alone formed a thin monolayer on top of the extra-cellular matrix (ECM), and were not affected by FGFR inhibition (PD173074). In the presence of stellate cells (PS1), there was a marked increase in cancer cell (COLO-357) number as well as invasion into the ECM (arrow head). This invasion was abrogated by FGFR inhibition (PD173074).Cytokeratin (green) and vimentin (red) staining, to delineate tumour and PS1 cells, respectively, confirmed a significant decrease in cancer cell invasion into the ECM upon FGFR inhibition (PD173074), compared to vehicle-treated (DMSO) cultures. Stellate cells appeared trapped within the overlying cell layer following PD173074 treatment and failed to migrate into the underlying ECM.Graph shows the reduction in cancer cell invasion into the ECM when cultures were treated with PD173074. Invading cohorts were analysed over twelve fields per organotypic gel. Each data point represents an average of invading cohorts across these twelve fields per gel. ***P = <0.0001. Student's *t*-test. Data summary represented by median ± interquartile range.There was no significant change in proliferative index (Ki67 staining) in organotypic cultures treated with PD173074 when PS1 and cancer cells were admixed, relative to when cancer cells were cultured alone. Student's *t*-test. Data summary represented by mean ± s.e.m. Data information: Scale Bar: 100 μm. Images are representative of at least nine organotypic gels for each condition from three independent experiments.

These data suggest a specific role for nuclear FGFR1 and FGF2 in driving PSC proliferation. In contrast to cancer cells, PSCs are exquisitely sensitive to FGFR inhibition, thus opening a new selective therapeutic avenue. The role of tumour-stroma cross-talk is recognised to play a role in PDAC progression (Vonlaufen *et al*, 2008; Froeling *et al*, 2009, 2011; Kocher *et al*, 2009; Olive *et al*, 2009). Since our observations suggest that targeting the stroma is an attractive option for therapy, we used a well-validated, pathologically-relevant PDAC model (Froeling *et al*, 2011) to specifically assess the interaction of cancer cells and stellate cells (Fig 5A).

### Blocking nuclear FGFR1 and FGF2 inhibits stellate cell invasion and abolishes cancer cell invasion in an organotypic model of PDAC

When cancer cells were grown alone in organotypic cultures, they failed to invade into the underlying stroma and showed no significant changes in cell proliferation or invasion upon FGFR blockade (PD173074, Fig 5B). However, when cancer cells were admixed with PS1 or primary PSCs there was a significant increase both in cancer cell number and in invasion of cancer and stellate cells into the matrix. This invasion was abrogated by FGFR blockade (Fig 5C and D, Supplementary Fig 9A–C), suggesting a pivotal role for PSCs in mediating pancreatic cancer cell invasion. There was no significant difference in cellular proliferation when organotypic cultures were treated with PD173074 or DMSO control, neither when they comprised cancer cells cultured alone, nor when cancer cells were admixed with PS1 cells (Fig 5E).

**Figure 6:**
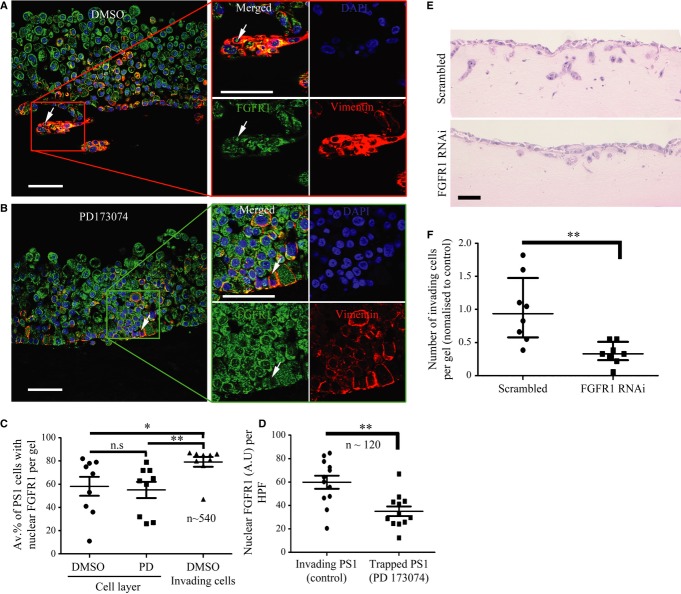
Differential FGFR1 localisation in stellate cells upon FGFR inhibition in 3D cultures. A–C The percentage of stellate cells (identified by vimentin stain: red) demonstrating nuclear FGFR1 (green) was significantly less in the stellate cells that failed to invade into the extra-cellular matrix) as compared to those invading (arrow in A) in vehicle-treated organotypic cultures. Upon FGFR inhibition (PD173074), stellate cells failed to localise FGFR1 to the nucleus and did not invade into the matrix (arrow in B, quantified in C). A total of four fields were counted per organotypic gel. Each data point represents an average of the percentage of stellate cells with nuclear FGFR1 over these four fields (total of 540 cells counted). C, *P = 0.0366, **P = 0.0092. D, **P = 0.0017. Student's *t*-test. Data summary represented by mean ± s.e.m. D Nuclear FGFR was also analysed in vimentin positive invading and ‘trapped’ stellate cells following PD173074 treatment, using Image J digital quantification (see Methods). Those cells that were able to invade (vehicle treated) showed significantly more nuclear FGFR1 than those cells that remained trapped in the cell layer following PD173074 treatment. Each data point represents the average nuclear FGFR1 intensity per field. Several fields were counted from three separate gels (a total of 120 stellate cells per condition were analysed). E, F Stellate cells were treated with FGFR1 or scrambled RNAi for 24 h before harvesting and culture in a mini-organotypic model admixed with COLO-357 cells in a 2:1 ratio. Gels were cultured for 7 days. H&E images show a significant reduction in total cell invasion when PS1 cells were depleted for FGFR1 compared to scrambled treated PS1 cells. Number of invading cells is quantified in F. **P = 0.0031. Student's *t*-test. Data summary represented by median ± interquartile range. Data information: Scale Bar: 100 μm. Images are representative of at least nine organotypic gels for each condition.

**Figure 7:**
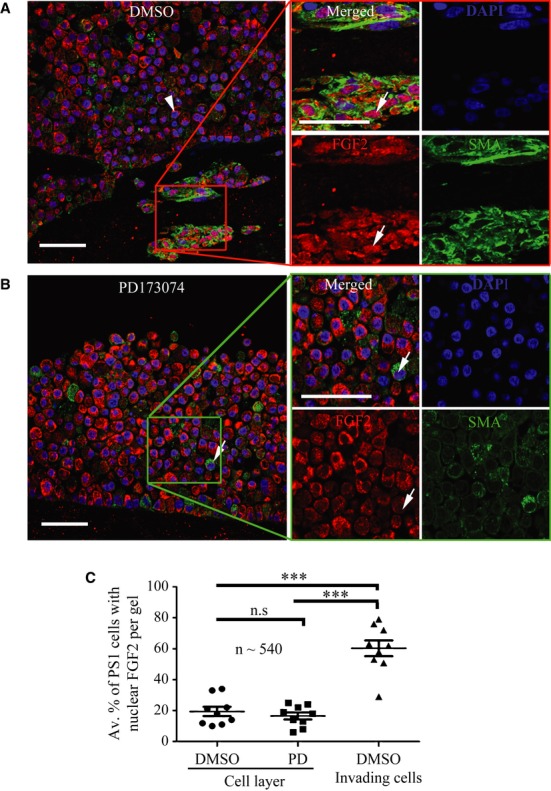
Differential FGF2 localisation in stellate cells upon FGFR inhibition in 3D cultures. In vehicle-treated organotypic cultures, FGF2 (red) was nuclear in a significant percentage of stellate cells (identified by αSMA stain: green, arrow in A, quantified in C) that invaded into the extra-cellular matrix, compared to those that remained within the admixed cell layer on top of the gel (arrow head in A). Upon FGFR inhibition (PD173074, 2 μM, 14 days), stellate cells failed to invade into the ECM. FGF2 was mainly cytoplasmic in these non-invading stellate cells (arrow in B). A total of four fields were counted per organotypic gel and each data point represents an average of percentage of stellate cells with nuclear FGF2 over these four fields (total of 540 cells counted). C, ***P < 0.0001. Student's *t*-test. Data summary represented by mean ± s.e.m. Data information: Scale Bar: 100 μm. Images are representative of at least nine organotypic gels for each condition.

Next we interrogated these physiomimetic cultures for localisation of FGFR1 and FGF2 in specific cellular compartments (within both cancer and stromal cells), upon PD173074 treatment. FGFR1 localised to the nucleus in the stellate cells invading into the matrix, whereas those stellate cells remaining juxtaposed to cancer cells showed less frequent nuclear localisation (Fig 6A–C). We also confirmed that PS1 cells that were able to invade into the matrix showed significantly more nuclear FGFR1, compared to PS1 cells remaining in the overlying cell layer, using digital quantification (Fig 6D). To confirm this phenomenon was specific to FGFR1 (PD173074 is a pan-FGFR inhibitor) we admixed PS1 cells, that had been treated with FGFR1 RNAi, together with COLO-357 cells in a 2:1 ratio and cultured on top of a mini-organotypic gel for 7 days. Knock-down of FGFR1 in stellate cells resulted in a significant reduction in cell invasion, compared to cultures generated with scrambled-treated PS1 cells (Fig 6E and F). In addition, there was a significant increase in the percentage of invading stellate cells with nuclear FGF2, compared to non-invading stellate cells (Fig 7A and C).

When treated with PD173074, PS1 cells were unable to invade into the extra-cellular matrix and FGF2 remained cytoplasmic (Fig 7A–C). Similar results could be demonstrated in organotypic cultures constructed with cancer-associated primary stellate cells obtained from patients (Supplementary Fig 9D). Furthermore, we confirmed in 2D culture that nuclear translocation of FGF2 and FGFR1 in stellate cells is regulated by factors secreted specifically by cancer cells rather than normal epithelial cells (Supplementary Fig 10).

### Nuclear FGF2 and FGFR1 at the invasive front of human PDAC

**Figure 8:**
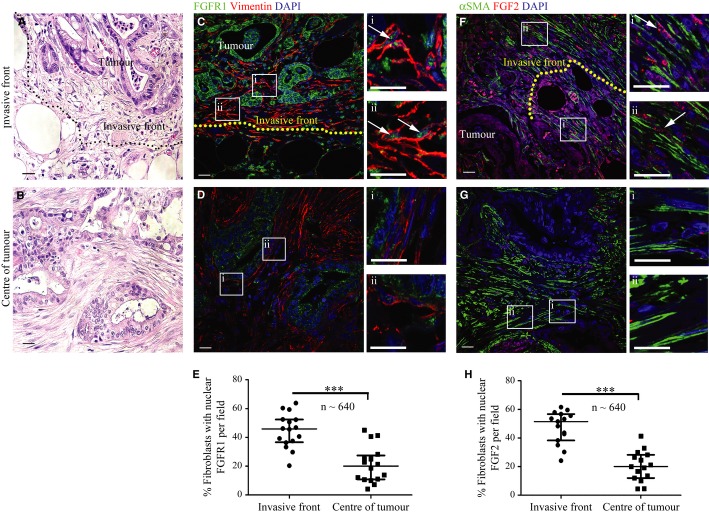
Fibroblasts at the invasive front of human PDAC show significantly more nuclear FGF2 and FGFR1. A, B H&E stained sections adjacent to those used for FGFR1 and FGF2 staining show the tumour invading into adipose tissue (A) or the central section of the tumour (B). C–E Fibroblasts (vimentin positive, red) invading adipose tissue (invasive front demarcated, C) in PDAC sections showed increased nuclear FGFR1 (green) relative to those at the centre of the tumour (D) (magnification of boxed areas, which represent stromal fibroblasts, are shown in Ci, Cii, Di and Dii). Quantification (E) of PDAC patient sections showed that a significantly higher number of fibroblasts at the invasive edge of the tumour (invading adipose, normal tissue or duodenum) had nuclear FGFR1, compared to those fibroblasts close to the centre of the tumour. E. ***P = 0.0001. Mann–Whitney *U*-test. Data summary represented by median ± interquartile range. F–H Staining of serial sections of the same tumour as in C, revealed that a significantly higher number of myo-fibroblasts (αSMA positive, green) invading adipose tissue in PDAC sections (F) showed nuclear FGF2 (red), compared to those at the centre of the tumour (G) (magnification of boxed areas, which represent stromal fibroblasts, are shown in Fi, Fii, Gi and Gii). Quantification is shown in (H). Each data point represents the percentage of fibroblasts (vimentin or αSMA positive) with nuclear FGFR1 or FGF2 per field. Several fields at the invasive front or centre of the tumour were quantified per patient. Stromal fibroblasts were analysed in four patients (*n* = 4640 fibroblasts at the invasive front and central tumour were counted in total).H. ***P < 0.0001. Mann–Whitney *U*-test. Data summary represented by median ± interquartile range. Data information: Scale Bar: 20 μm.

In agreement with our *in vitro* data, examination of human PDAC (whole tissue sections rather than TMAs) showed a significant increase in the percentage of fibroblasts demonstrating nuclear FGFR1 and FGF2 at the invasive front (invading into adipose tissue, duodenum or normal pancreatic tissue) as compared to those within the centre of the tumour (Fig 8). Taken together, these data suggest strongly that nuclear translocation of FGFR1, and consequently FGF2, facilitates stellate cell proliferation and motility. Upon effective blockade of nuclear FGFR1 signalling, we can abolish cancer cell invasion.

## Discussion

Our studies identify a novel role by which nuclear FGFR1 and FGF2 may regulate PSC behaviour in PDAC. In addition to its classical role as a receptor-binding growth factor, FGF2 can be targeted to the nucleus, regulating cellular proliferation. Understanding the role of intracrine FGF2 in PDAC has been facilitated by the isolation, and creation, of PSC cell lines (Apte *et al*, 1998; Froeling *et al*, 2009; Li *et al*, 2009; Erkan *et al*, 2012). These cells are thought to serve as key drivers in the pathobiology of PDAC stroma, where they switch from a quiescent to myo-fibroblastic state (Vonlaufen *et al*, 2008; Erkan *et al*, 2012; Tian *et al*, 2012). We show that both FGF2 and FGFR1 co-localise to the nucleus exclusively in PSCs *in vivo* (human PDAC) and *in vitro*.

Confocal analysis revealed co-localisation of FGFR1 and FGF2 at distinct nuclear speckles (Peng *et al*, 2002; Stachowiak *et al*, 2003), sites of RNA polymerase II mediated transcription and co-transcriptional pre-mRNA processing (Crispino *et al*, 1994), suggesting that nuclear FGFR1 and FGF2 regulate transcription of genes involved in proliferation. Indeed, FGFR1 can bind effectively to all isoforms of FGF2; the LMW isoform, which is generally extracellular, as well as the predominantly nuclear HMW isoforms. Studies have shown that HMW FGF2 may be required to regulate the nuclear entry and mobilisation of FGFR1, facilitating the interaction between FGFR1 and gene promoters and other nuclear proteins, thereby regulating transcription (Dunham-Ems *et al*, 2009).

In addition to FGF2 localising at speckles with FGFR1, we observed FGF2 in the nucleolus, a major site of ribosomal synthesis. FGF2 localisation to the nucleolus was first identified in adult bovine aortic cells, suggesting a role in driving quiescent cells into a proliferative state (Bouche *et al*, 1987). Nuclear localisation of FGF2 in these studies correlated with stimulation of transcription of ribosomal genes, during transition from G0 to G1 phase of the cell cycle, and increased expression of the major non-histone nucleolar protein, nucleolin, which has a key role in ribosomal transcription (Bugler *et al*, 1982). FGF2 also had a direct effect on the enhancement of RNA polymerase I activity in nuclear extracts isolated from quiescent cells, implying a mitogenic role for nuclear FGF2 (Stachowiak *et al*, 1994; Joy *et al*, 1997; Peng *et al*, 2002). Therefore, it is possible that FGFR1 and a pool of FGF2 may influence distinct nuclear functional domains in PSCs. Studies have shown that FGFR1 interacts more with HMW FGF2 than LMW FGF2 (Dunham-Ems *et al*, 2009). Furthermore, HMW FGF2 can decrease the mobility of nuclear FGFR1 following stimulation, to facilitate the interaction of the receptor with gene promoters and other nuclear proteins such as CREB-binding protein (CBP), thus influencing transcriptional activity (Dunham-Ems *et al*, 2009). Thus, the nuclear localisation of FGF2 within distinct regions of the nuclei of PSCs may be driving distinct biological effects either with or without its receptor. The concept that nuclear FGFR1 and FGF2 may have a functional relationship exclusively in stellate cells was strengthened by the observation that, upon FGF2 knock-down, FGFR1 failed to translocate to the nucleus. However, no effect was observed on nuclear FGFR1 in cancer cells, which do not have nuclear FGF2. Chemical inhibition of FGFR signalling prevented nuclear translocation of FGFR1 and FGF2 in PSCs, suggesting that FGF2 driven FGFR1 signalling is important for nuclear translocation within PSCs. Following treatment of PSCs with recombinant FGF2, a known ligand of FGFR1 (Zhang *et al*, 2006), we observed a dramatic localisation of both FGFR1 and FGF2 to the nucleus, suggesting that FGFR activation is important for nuclear FGFR1 and FGF2 trafficking in PSCs. Indeed, abolishing FRS2 in PSCs also had a profound effect on nuclear FGFR1 and FGF2. These data, taken together with recent independent observations (Bryant *et al*, 2005; Chioni & Grose, 2012), suggest that receptor activation is required for internalisation and translocation. Furthermore, since FGF2 is secreted by PSCs but not cancer cells, it may fuel an FGFR1 autocrine signalling loop in these cells, stimulating nuclear localisation of FGFR1 and FGF2.

Silencing FGF2 or FGFR1, or blocking FGFR signalling with PD173074, in PSCs resulted in significant reduction in cell proliferation. These effects were not apparent in PDAC cell lines, which did not display nuclear FGF2, suggesting that nuclear FGFR1 and FGF2 may co-activate genes involved in cellular proliferation exclusively in PSCs. Indeed, nuclear FGFR1 has been shown to regulate *c-Jun* and *Cyclin D1* in human glial cells (Reilly & Maher, 2001). Blocking nuclear FGFR1 and FGF2 in PSCs, using PD173074, correlated with a G1 cell-cycle block and a significant reduction in cyclin D1 expression. Activation of cyclin D1 by nuclear FGFR1 and FGF2 may drive entry into the cell cycle, as has been shown in neuronal cells (Joy *et al*, 1997; Stachowiak *et al*, 1997a). Moreover, nuclear FGFR1 has been shown to regulate *FGF2* gene expression by indirectly activating the *FGF2* promoter (via cAMP and PKC dependent signalling pathways) (Peng *et al*, 2001). We also observed a significant reduction in FGF2 expression following FGFR1 knock-down exclusively in PSCs, with no effect in PDAC cells, providing further evidence for the critical role of nuclear FGFR1-driven proliferation in PSCs.

Blocking FGFR signalling in our organotypic model resulted in striking effects on cancer cell and PSC invasion. Following treatment with PD173074, there was a significant block of invasion of cancer cells into the ECM, and it was apparent that PSCs were ‘trapped’ within the overlying cell layer. In vehicle-treated organotypic cultures, there was a significant increase in the number of PSCs with nuclear FGFR1 and FGF2 that were able to invade into the ECM. Following FGFR blockade, PSCs remaining in the cancer cell layer displayed mainly cytoplasmic staining for both FGFR1 and FGF2. Furthermore, flexibility of the organotypic system allowed us to use PSCs that had been treated with FGFR1 RNAi to demonstrate that this regulation of stellate cell invasion, and modulation of cancer cell behaviour, is specific to FGFR1.

These results suggest that nuclear FGFR1 and FGF2 may have a profound effect on PSC invasion, which in turn mediates cancer cell invasion. Further studies will investigate changes in PSC gene expression in appropriate physiologically relevant 3D culture conditions, in the presence of cancer cells, to define the exact role of paracrine and autocrine FGF signalling. Examination of PDAC sections revealed that a significant percentage of myofibroblasts at the invading tumour front had nuclear FGFR1 and FGF2, compared to myofibroblasts in the central core of the tumour. This further emphasises the possible role of nuclear FGFR1 and FGF2 in driving PDAC tumour invasion.

Taken together, we conclude that the striking anti-invasive effect, seen when organotypic cultures were treated with PD173074, was a consequence of a change in the cellular microenvironment provided by PSCs. *In vivo,* the stroma is now appreciated as a major driver in promoting the aggressiveness of PDAC and makes up 80% of the tumour volume (Froeling *et al*, 2011). The presence of PSCs in orthotopic models of PDAC increases distant spread of the tumour (Vonlaufen *et al*, 2008) with PSCs co-migrating with cancer cells to distant sites, likely aiding in the translocation of the tumour in the new microenvironment. We propose that preventing nuclear FGF/FGFR mediated proliferation in PSCs leads to disruption of the tumour microenvironment, preventing pancreatic cancer cell invasion, thus identifying a novel therapeutic approach targeting within the stroma of PDAC.

## Materials and Methods

### Patient samples

Ethically approved human PDAC samples were arranged in tissue micro-array as described before (Froeling *et al*, 2009; Kocher *et al*, 2009).

### Culture conditions

Pancreatic cancer, stellate (primary PSCs and an immortalised pancreatic stellate cell line, PS1) and normal epithelial cells were cultured as adherent monolayers in sterile tissue culture flasks as described before (Froeling *et al*, 2009; Kocher *et al*, 2009; Li *et al*, 2009). Finely sliced fresh human pancreatic tissue, obtained in an ethically approved manner, was used to derive primary pancreatic stellate cells as described before (Bachem *et al*, 2005). Cell numbers used for different assays are displayed in Supplementary Table 1.

### Antibodies

The antibodies, along with dilutions used, are listed in Supplementary Table 2.

### Inhibitors and RNAi

PD173074 (Sigma-Aldrich, Dorset, UK), a potent, cell permeable and ATP competitive inhibitor of FGFR (Mohammadi *et al*, 1998b) was dissolved in DMSO (stock: 20 mM) to be used at a 2 μM final concentration. Equivalent volumes of vehicle (DMSO) served as control.

Cells were transfected with a pool of siRNA oligonucleotides (Supplementary Table 3, Dharmacon, CO, USA) targeting Human FGF2, FGFR1 or FRS2 at a final concentration of 10 nM using INTERFERin™ (Polyplus, Illkirch, France), with non-targeting siRNA as a control. Knock-down efficiency was confirmed by Western blot (72 h post-transfection). Subsequently, PS1 cells treated with FGFR1 RNAi (24 h) were harvested, admixed with COLO 357 cancer cells in a 2:1 ratio and cultured in a mini-organotypic model for 7 days.

### Organotypic cultures

As described before (Froeling *et al*, 2009, 2011; Kadaba *et al*, 2013), either cancer cells alone (control) or cancer cells admixed with PS1 cells in a 1:2 ratio, were plated onto gels composed of 75% Collagen type I (3 mg/ml, 5.25 volumes) and 25% Matrigel (1.75 volumes; both BD Biosciences MA, USA), 1 volume 10X DMEM, 1 volume 1X DMEM (PAA, Yeovil, UK) and 1 volume of filtered FBS (PAA). The next day, the gels were lifted onto a metal grid and fed from below with medium supplemented with PD173074 (2 μM) or Dimethyl sulfoxide (DMSO; control). Mini-organotypics were constructed using primary PSCs or PS1 cells treated with FGFR1 or scrambled RNAi (24 h treatment) in 0.4 μM Transwell inserts (Corning, NY, USA) with less cells as dictated by limited availability of primary cells. Normal medium or medium supplemented with either PD173074 (2 μM) or DMSO (vehicle), was changed on alternate days and gels were harvested at 14 days (or 7 days for mini-organotypic cultures), fixed in 10% neutral buffered formalin, bisected and embedded in paraffin. Cell invasion in the organotypic cultures was calculated by determining the number of cohorts (two or more cells) of cancer cells invading into the extra-cellular matrix. Twelve high power fields (HPF) were counted for each organotypic gel and averages of these fields were plotted, each represented by one data point. Nine gels were analysed from three separate experiments. Alternatively, to quantify invasion in the mini-organotypic model in which PS1 cells were treated with FGFR1 RNAi, total cell number invading per gel was plotted.

### FGF2 stimulation assays

For inhibitor experiments, cells were treated for 1 h with FGFR inhibitor PD173074 (2 μM) before stimulation with 100 ng/ml recombinant FGF2 (PeproTech, London, UK) and 300 ng/ml heparin sodium salt (Sigma-Aldrich), for different time points. Similarly, conditioned media (serum-free) from normal or cancer cells were added.

### Cell growth assay

Cells, exposed to PD173074 (2 μM) or DMSO (control) for designated time-points after treatment, were detached with trypsin-EDTA and counted with a Casy counter (Scharfe, Reutlingen, Germany). Cell counts were normalised to the untreated cell number 24 h after plating. Each experiment was carried out in triplicate and repeated on three separate occasions. FACS based cell-cycle analysis was performed after 48 h of treatment using propidium iodide staining as described before (Froeling *et al*, 2011). Ki67 staining was also used to identify proliferating cells. Each data point plotted represents the percentage of cells positive for Ki67 per field. Multiple fields were taken per experiment. The total number of cells analysed to obtain this percentage was recorded in the figure.

### Western blot analysis

Cells were lysed, protein samples prepared and run on polyacrylamide gels as described previously (Jarosz *et al*, 2012). For conditioned medium Western blot analysis, cells were cultured in serum-free medium for 12 h, after which time the medium was collected, filtered, concentrated 20× using NMWL 3000 centrifugal filter units (Millipore, Watford, UK) according to the manufacturer's instructions, and boiled with sample buffer. Serum-free medium was used as a negative control.

For cell fractionation, experiments were performed according to manufacturer's instructions (Nuclear Extraction kit, Imgenex, CA, USA) and fraction purity was confirmed (Lamin A/C and Tubulin antibodies for the nuclear and cytoplasmic fractions respectively).

Densitomitry of bands was performed using Image J software 1.429 (National Institute of Health, USA). Respective protein band densities were normalised to the loading control detected on the same membrane.

### Immunofluorescence

For immunofluorescence, cells were fixed with 4% formaldehyde, permeabilised with 0.1% saponin/PBS and blocked with 6% BSA (1 h), followed by incubation with primary antibody (room temperature, 1 h) and then appropriate fluorescently labelled-secondary antibody (room temperature, 1 h). Nuclei were stained with 4′,6-diamidino-2-phenylindole (DAPI). For paraffin embedded organotypic gels and patient tissues, 4 μm sections were dewaxed and rehydrated through a graded ethanol series and antigens were retrieved by boiling sections in 10 mM citrate buffer (pH 6.0, for 20 min). Sections were blocked, incubated with primary antibody (overnight, 4°C), followed by secondary antibody and DAPI as above. Negative controls were incubated with isotype-specific immunoglobulins at matching dilution.

Total FGF2, nuclear FGF2 and FGFR1 levels were quantified with Image J software 1.429. Images were taken at ×630 magnification, and the area of red or green fluorescence within the region of interest (DAPI staining) was determined. Thresholds were set and kept constant for all images analysed. Multiple fields (at least 3) were taken per experiment and the total cells per field were analysed. An average of total, nuclear FGFR1 or FGF2 per field was plotted. All experiments were carried out on three separate occasions. The total number of cells analysed is recorded in each figure.

To analyse the percentage of cells with nuclear FGFR1 and FGF2 in organotypic sections, multiple fields per gel were analysed. The average percentages of PS1 cells with nuclear FGFR1 or FGF2, across these fields, were plotted and are shown by each graphical data point. Nine gels were analysed in total, from three separate experiments. In order to analyse the percentage of nuclear FGFR1 and FGF2 positive fibroblasts, at the invasive front or in the centre of the tumour in tissue sections, several fields per section per patient (four patients) were analysed. Each data point is representative of one HPF. The total number of fibroblasts analysed for all the patients is recorded in the figure.

**The paper explained**

**Problem**

Patients who are diagnosed with pancreatic ductal adenocarcinoma (PDAC) face a dismal prognosis. One reason for this is the dense stroma that is a characteristic of PDAC, which may preclude drugs from accessing the tumour cells. Pancreatic stellate cells (PSCs) are the key cell responsible for desmoplasia in PDAC and it is becoming clear that they are a promising target for therapy. FGFs and their receptors are frequently over expressed in pancreatic cancer and FGF2 over-expression correlates with poor patient outcome. It is already known that FGF2 and full-length FGFR1 localise to the nucleus in neuronal cells, while a cleaved FGFR1 form localises to the nucleus in breast cancer cells and correlates with a more metastatic phenotype. Previously, FGF2 has been shown to localise to the nucleus in pancreatic cancer but not normal pancreatic tissue, suggesting nuclear FGF2 may play a role in PDAC progression, however the function of nuclear FGF2 in PDAC is not understood.

**Results**

Nuclear FGFR1 and FGF2 are apparent in the stromal fibroblasts at the invasive front of human pancreatic cancer. *In vitro* FGFR1 and FGF2 co-localise to the nucleus in pancreatic stellate cells but not pancreatic cancer cells and are essential for proliferation and invasion. Blocking nuclear FGFR1 and FGF2 results in a significant reduction in proliferation of pancreatic stellate cells and has a significant effect on invasion of pancreatic cancer cells in a 3D organotypic model of pancreatic cancer.

**Impact**

We have shown that targeting nuclear FGFR1 and FGF2 has a specific effect on PSC proliferation. As a consequence, the tumour microenvironment provided by PSCs is disrupted, and cancer cell invasion is prevented. Specific stromal targeting therapy may modify PDAC patient survival.

### Co-localisation

Double-stained images were taken using a confocal laser scanning microscope (Zeiss LSM 710, Carl Zeiss, Germany) and thresholds for each channel of interest were set to correct for background fluorescence. Co-localisation of two proteins appeared as white pixels (Supplementary Fig 1). Nuclear FGFR1 and FGF2 co-localisation in PDAC tissue microarrays (TMAs) was quantified by counting the total number of stromal fibroblasts and cancer cells per core (one core per patient, 46 patients were scored) and then assessing the percentage of those cells that showed co-localisation of FGFR1, FGF2 and DAPI (white pixels) with constant pre-set thresholds. For patients in whom both FGFR1 and FGF2 were scored (36 patients) correlation between the presence of FGF2 and FGFR1 in the nuclei of stromal fibroblasts was assessed. The same technique was used for *in vitro* cellular co-localisation of FGFR1 and FGF2.

### Statistical analysis and quantification

All quantitative data are presented with respective statistical tests dependent on normality distribution and significance was defined as *P* < 0.05 as analysed in Prism v 5.03 (GraphPad, La Jolla, CA, USA). Unless otherwise stated, all experiments were performed independently a minimum of three times, each time in triplicate.
