# Supplementary material for: Nuclear translocation of FGFR1 and FGF2 in pancreatic stellate cells facilitates pancreatic cancer cell invasion
Source: EMBO Mol Med. 2014 Feb 6;6(4):467–81. doi: 10.1002/emmm.201302698 (PMC3992074; doi:10.1002/emmm.201302698)
Supplement: Supplementary file 1 [file emmm0006-0467-sd1.pdf]

EMBO Molecular Medicine

Nuclear translocation of FGFR1 and FGF2 in pancreatic stellate cells facilitates pancreatic cancer cell invasion

Stacey J Coleman, Athina-Myrto Chioni, Mohammed Ghallab, Rhys K Anderson, Nicholas R Lemoine, Hemant M Kocher, Richard P Grose

Corresponding author: Richard Grose, Queen Mary University of London

Review timeline:

Submission date:	05 March 2013
Editorial Decision:	24 April 2013
Additional Author Correspondence :	29 April 2013
Additional Editorial Correspondence:	02 May 2013
Additional Author Correspondence :	02 May 2013
Additional Editorial Correspondence:	03 May 2013
Revision received:	14 November 2013
Editorial Decision:	05 December 2013
Revision received:	17 December 2013
Accepted:	20 December 2013

Transaction Report:

(Note: With the exception of the correction of typographical or spelling errors that could be a source of ambiguity, letters and reports are not edited. The original formatting of letters and referee reports may not be reflected in this compilation.)

Editor: Roberto Buccione

1st Editorial Decision 24 April 2013

Thank you for the submission of your manuscript to EMBO Molecular Medicine. We have now heard back from the three Reviewers whom we asked to evaluate your manuscript.

You will see that all three Reviewers are generally supportive of you work although Reviewer 1, in particular, expresses significant concerns that prevent us from considering publication at this time. I will not discuss each point in detail as they are clearly stated.

Reviewer 1 points to a number of experimental issues that require your action. S/he is especially concerned that key experiments lack sufficient controls and that the conclusions rely too heavily on antibody staining and imaging. S/he also feels that about the mechanistic aspects of the study are insufficiently developed. Reviewer 1 suggests a number of experimental approaches to improve the manuscript in these aspects.

Reviewer 2 also notes some lack of mechanistic depth, in particular concerning downstream signalling from nuclear FGFR1 and would also like to see whether FRS2 depletion would still allow for FGF2/FGFR1 translocation to the nucleus.

Reviewer 3 is especially concerned that imaging and statistical analyses are not optimal and need to be significantly improved and lists a number of essential points that require your action. S/he also notes that it would be desirable but not essential to digitally quantify nuclear FGFR1 in the

organotypic assays. Nevertheless, I would actually strongly encourage you to perform this analysis to improve the quality and stringency of your data.

As one Reviewer noted, this field is indeed still controversial and I strongly concur that further analysis is required to reduce vulnerability and uncertainty. In this respect, Reviewer 1's concerns are especially punctual.

Considered all the above, while publication of the paper cannot be considered at this stage, we would be prepared to consider a revised submission, with the understanding that the Reviewers' concerns must be fully addressed, with additional experimental data where appropriate and that acceptance of the manuscript will entail a second round of review.

Please note that it is EMBO Molecular Medicine policy to allow a single round of revision only and that, therefore, acceptance or rejection of the manuscript will depend on the completeness of your responses included in the next, final version of the manuscript.

As you know, EMBO Molecular Medicine has a "scooping protection" policy, whereby similar findings that are published by others during review or revision are not a criterion for rejection. However, I do ask you to get in touch with us after three months if you have not completed your revision, to update us on the status. Please also contact us as soon as possible if similar work is published elsewhere.

I look forward to seeing a revised form of your manuscript as soon as possible.

***** Reviewer's comments *****

Referee #1 (Remarks):

The manuscript entitled "Nuclear translocation of FGFR1 and FGF2 in pancreatic stellate cells facilitates pancreatic cancer cell invasion" by Stacey J Coleman, et al., reports that FGF2 and FGFR1 localize to the nucleus in activated stellate cells (PSC) of human pancreatic cancer, but not in cancer cells. Inhibiting FGFR1 and FGF2 in PSCs resulted in reduced cell proliferation. PSCs with nuclear FGFR1 and FGF2 pomote cancer cells to invade the underlying extra-cellular matrix. FGFR inhibition prevented PSC as well as cancer cell invasion in the coculture system. FGFR inhibition resulted in cytoplasmic localization of FGFR1 and FGF2, abrogation of nuclear FGFR1 and FGF2 in PSCs and abolished cancer cell invasion. These findings suggest that disruption of nuclear FGF/FGFR mediated proliferation and invasion in PSCs can be a novel therapeutic approach for preventing pancreatic cancer metastasis. However, most of the conclusions are descriptive and correlation, without mechanistic studies. How FGF2 and FGFR1 are translocated to the nucleus is not addressed. Furthermore, localization of both FGF2 and FGFR1 are only evidenced by antibody staining, which is not convincing. The specificity of the antibodies is not well validated although similar experiments have been reported by the same lab. Other methods are needed to confirm the nuclear localization of the two molecules. In addition, too many supplementary data make it difficult to read the manuscript.

Specific points:
1. Fig. 1 and others: Nuclear localization data are not convincing. ? Preabsorbed antibody with FGFR1 or FGF2 are good negative controls, which will at least confirm that the staining are not just non-specific staining.
2. Figure 2, why FGF2 also go to nuclear independent of the receptor? What is the function of receptor-independent function of FGF2. If FGF2 or FGFR2 functions as a transcription factor, then chip-seq will be a good way to identify the responsive elements of the two molecules.
3. Fig. 2E, fgf2 treatment should be done to see it exogenous FGF2 can induce FGFR1 nuclear localization.
4. Fig. 2E need to show that the activity of FGFR1 is specific, other three non-FGFR1 expressing cells should be used as a control, the same is needed for Fig. 5.
5. Fig. 2J, why only some cells are inhibited? Different dosages should be used to show the activity

is dosage dependent.
6. Fig. 3A, B, why fgf2 does not colocalized with sc35? Does it bind to fgfr1?
7. Fig. 6 ac, why fgfr1 is punctate stained and fgf2 is diffusely stained. Do they colocalized?
8. Fig. 8, need high resolution and high magnification pictures for evaluation.

Referee #2 (Remarks):

These authors uncovered that FGF2 and full length FGFR1, in activated desmoplastic stroma of the pancreatic ductal adenocarcinoma, localize to the nucleus of the stellate cells but not in cancer cells. Moreover the nuclear localization of FGF2/FGFR1 is crucial for the stroma cells proliferation and migration as well as the cancer invasion. Further, these findings indicate for novel prospective drug targets that may result in preventing pancreatic cancer cells invasion.
Although translocation of full length growth factor receptors in to the nucleus (and not only to the lysosome) it is still a controversial issue, the quality of the presented data and cited literature makes me convinced.
In my opinion the studies are well designed, carefully performed and the data are solid. The discovery is significant and has a potential to impact on drug screening. On the other hand this finding could also have significant impact on understanding FGF/FGFR signal transduction in general.

However, there are two questions the study did not clearly address:

(1). Authors did not touch the interesting matter concerning the downstream signaling of nuclear FGFR1. They just restricted their comments that the nuclear receptor-induced effects in PS1 cells are sensitive to PD173074 treatment or FGFR1 depletion and that a treatment with the inhibitor resulted also in a significant reduction in nuclear FGF2 (both isoform) as well as full length FGFR1. The lack of a piece of rational speculations on this makes the story more difficult to "digest".

(2). I am curious if FGF2/FGFR1 will still be translocated to the nuclear compartment under FRS2 depletion?

Referee #3 (Remarks):

The authors present an interesting and important manuscript where they claim a role for both nuclear FGF2 and FGFR1 in pancreatic stellate cells (PSCs) in promoting pancreatic ductal adenocarcinoma (PDAC) cancer cell invasion. The authors look at nuclear colocalization of FGF2 and FGFR1 alongside markers of PDAC and activated PSCs in pancreatic tumors via TMAs and in a series of PDAC cell lines and immortalized and primary PSCs. Knockdown of FGF2 (and PD173074 treatment) results in reduced nuclear expression of the FGFR1 and visa versa, but only in PSC cells and not PDAC cells. Moreover FGF2/FGFR1 knockdown or PD inhibition results in reduced proliferation of PSCs but not PDAC cells. Most importantly the authors functionally tested the role of FGF2/FGFR1 in an organotypic model of PDAC. When cancer cells were grown alone they did not invade into the underlying matrix whereas when PSC were added to the cancer cells there was considerable invasion into the underlying matrix which was blocked by PD173074. This inhibition of invasion was then correlated with nuclear localization of FGFR1 and FGF2 and was similar when immortalized PSCs were used as well as cancer associated primary PSCs were used. The authors then confirmed this in whole tissue sections from patient samples where there was a significant increase in the percentage of fibroblasts showing nuclear FGF2/FGFR1 at the invasive front compared to the core. Hence, the authors have potentially identified a specific therapeutic target in treatment of pancreatic cancer.
This is a significant and novel finding that expands our understanding of FGF signaling in PDAC. The discussion adequately addresses earlier literature and stands out from other papers in using colocalization imaging across a combination of cell lines, organotypic cultures utilizing cancer cells

and primary patient pancreatic stellate cells and FFPE patient samples to examine the role of FGF2/FGFR1 in the nuclei of pancreatic stellate cells. It also employed both knockdown and pharmaceutical inhibition. Overall this work has taken a novel approach to understanding the important role of FGF signaling in PDAC that may lead to a novel treatment however additional methodological data regarding imaging quantitation is required.

Major issues:

In general insufficient methodological data was provided for the quantitation of the imaging e.g. more explanation of the number of cells examined in each condition needs to be supplied for each data point provided in the graphs. In the methods the authors state that 3 biological replicates were performed in triplicate however some graphs have 9 data points and others have more which are not explained. In each case the authors but do not state how many images/cells were examined in each replicate.
1. In figure 1, the relationship between cells counted and the correlation within each patient is not clear. In Figure 1B and 1D, What does n=46 refer to? Number of cells from each patient examined? In figure 1F there appears to be 36 data points, was this all patients on the TMA?
2. The graph in Figure 2A was a little confusing, would the X-axis be better labelled as colocalization rather than PS1. In Figure 2D is each data point 1 cell (presumably not) or the average of a replicate experiment including multiple cells/images. For figures 2H-L, there are 12 data points but no explanation as to what each data point is summarizing, one image or the average of multiple images from one replicate experiment? Please add explanation to methods.
3. For Figure 3C-H and 4B, the authors graph % Ki67 cells but do not state how many cells were examined to get this percentage? In figure 6C and 7C, the authors graph % of PS cells with nuclear FGFR1/FGF2, again how many cells were analysed in each replicate experiment to obtain percentage. Similar issue with Figure 8C.
4. In figure 5D, can the authors add how many HPF were counted in each replicate experiment to the methods?
5. In relation to the organotypic assays, figure 7 is convincing. Stellate cell nuclear FGF2 localisation is very clear in the control, as is the failure to localise in the nucleus, in the presence of inhibitor. However, figure 6, is not convincing. FGFR1 looks to be no different in control and treatment conditions in the vimentin positive-stellate cells. This may be due to the choice of representative image. Alternatively, given the importance of the organotypic model system employed in validating the proposed mechanism, the paper would be strengthened by digital quantitation of the nuclear FGFR1 in invasive cells versus "trapped PSCs" following PD173074. For example, could authors apply thresholding to quantify nuclear FGFR2 in the vimementin positive cells.
6. In the organotypic culture assays, the authors only use the inhibitor which does not differentiate between FGF/FGFR family members. The manuscript would be strengthened by depleting stellate cells of FGFR1 only and show that invasion does not occur in these organotypic assays.
Addressing comments 1-4 are essential, addressing comment 5 is highly desirable.

Minor Issues:

Figure legends were a little confusing, often grouping figures A-D, to describe something. The authors should consider expanding the figure legends to describe each item in the figure.

Additional Author Correspondence 29 April 2013

Many thanks for your email - we were delighted to read the very complimentary and constructive comments from the three reviewers and are in the process of addressing them all in detail. As only one round of resubmission is allowed, at this stage I would just like to clarify a couple of comments from reviewer 1. I am happy to discuss this on the phone but perhaps email is sufficient?

Reviewer 1 Specific points:
1. Fig. 1 and others: Nuclear localization data are not convincing. ? Preabsorbed antibody with FGFR1 or FGF2 are good negative controls, which will at least confirm that the staining are not just non-specific staining.

Regarding nuclear localisation, not only do we have convincing confocal z-stacking data (Fig 2A) and line plotting (Fig 3B), but also subcellular fractionation by western blotting (Fig 2F and 2M; Sup Fig 5A). To my mind these are gold standard methods of determining nuclear localisation. Clearly, they all rely on antibody specificity, so we have included already in the paper multiple independent examples of western blotting and immunofluorescence for both FGFR1 and FGF2 on cells treated with RNAi to each, including the whole of Sup Fig 4, which includes western blot and IF data for knockdown of both proteins in stellate and cancer cell lines, but also:
FGF2: Figs 2C and 3C for FGF2 RNAi in stellate cells; Sup Fig 8A and D for cancer cells.
Specific RNAi knockdown by subcellular fractionation (Fig 2F, Sup Fig 5A)
FGFR1: Fig 2G for FGFR1 RNAi in stellate cells.

We will also include controls with our FGF2 antibody preabsorbed against recombinant FGF2, and santa cruz anti-FGFR1 western blotting antibody preabsorbed against the immunising peptide. Unfortunately this is not possible for the Abcam FGFR1 antibody that we use for immunofluorescence, as we don't have access to recombinant receptor protein or the immunising peptide. As acknowledged by the reviewer, we have already provided thorough characterisation of this antibody in a previous publication (Chioni and Grose, 2012, J Cell Biol.197(6):801-17).

4. Fig. 2E need to show that the activity of FGFR1 is specific, other three non-FGFR1 expressing cells should be used as a control, the same is needed for Fig. 5.

We simply don't understand what this point is asking for? We are showing decreased nuclear FGFR1 upon FGF2 knockdown. Which other three non-FGFR1 expressing cells? For Figure 5 we aim to address this by generating cultures with PS1 cells that have been treated with FGFR1 RNAi, as requested by Reviewer 3 pt 6.

We would be most grateful for clarification on whether these responses would be sufficient to address these particular two comments. All the other comments are very clear and we will address thoroughly.

Many thanks for any advice on this.

Additional Editorial Correspondence 02 May 2013

Thank you for your message and apologies for not being able to reply sooner.

Regarding the first point, I think that your strategy for dealing with the first point you mention is quite satisfactory. As for the second, I will contact the Reviewer to ask him/her to elaborate a little and get back to you.

Additional Author Correspondence 02 May 2013

Thanks very much for getting back to us. That's great about our first point and we look forward to the further clarification of the second.

Additional Editorial Correspondence 03 May 2013

I have now heard from the reviewer on the points we discussed.

Regarding the first, s/he is sorry for the confusion and means is that s/he would like to see is "a valid control showing that nuclear staining is true since the antibody may not be very specific and may provide misleading results." My impression is that the explanation/further data you plan to provide should be satisfactory, but we shall have to see what the Reviewer thinks.

As for the second, on the organotypic cultures, s/he states that your proposed strategy is fine "as long as the authors can provide a convincing negative control I think I will be satisfied".

I hope this helps

1st Revision - authors' response 14 November 2013

Section A. Response to Editor's comments

1. *Reviewer 1 points to a number of experimental issues that require your action. S/he is especially concerned that key experiments lack sufficient controls and that the conclusions rely too heavily on antibody staining and imaging. S/he also feels that about the mechanistic aspects of the study are insufficiently developed. Reviewer 1 suggests a number of experimental approaches to improve the manuscript in these aspects.*

We appreciate the comments from Reviewer 1. We have addressed their concerns comprehensively.

a) We have demonstrated multiple independent examples of antibody specificity after Western blotting (as well as sub-cellular fractionation) and immunofluorescence subsequent to either FGFR1 or FGF2 RNAi in different cell lines (improved Figures 2 and 3 and Supporting Information Figures 4 and 8).
b) Nuclear FGFR1 and FGF2 activity shows a dose dependent response to the FGFR inhibitor PD173074 (new data in Supporting information Figure 5B) and that FGF2 stimulation induces a rapid accumulation of nuclear FGFR1 and FGF2 in stellate cells (new data in Supporting Information 6G).
c) The functional relevance of these findings is stressed in Figure 6. Independently, both chemical and RNAi-mediated ablation of FGFR1 in pancreatic stellate cells causes reduction in cancer cell invasion in the physio-mimetic organotypic model (new Figure 6E,F).

The experiments stemming from these excellent suggestions/criticisms add significant weight to our findings. Specific concerns are addressed in Section B: Reviewer 1.

2. *Reviewer 2 also notes some lack of mechanistic depth, in particular concerning downstream signalling from nuclear FGFR1 and would also like to see whether FRS2 depletion would still allow for FGF2/FGFR1 translocation to the nucleus.*

We have now included new data in Supporting Information Figure 6 A-F, demonstrating that depletion of FRS2 (RNAi) has a profound effect on nuclear FGFR1 and FGF2; thus, supporting our hypothesis.

3. *Reviewer 3 is especially concerned that imaging and statistical analyses are not optimal and need to be significantly improved and lists a number of essential points that require your action. S/he also notes that it would be desirable but not essential to digitally quantify nuclear FGFR1 in the organotypic assays. Nevertheless, I would actually strongly encourage you to perform this analysis to improve the quality and stringency of your data.*

The reviewer's comments have strengthened the presentation of our existing dataset. The updated Figure 1 (panel B, D); Figure 2 (panel D, E, H, I, K, L); Figure 3 (panel D, G); Figure 4 (panel B); Figure 5 (panel D); Figure 6 (panel A-E); Figure 7 (panel C) and Figure 8 (panel E, H), Figure

Legends and Methods section ensure experimental clarity (automated, unbiased digital analysis) and statistical rigour (multiple technical and biological repeats).

As one Reviewer noted, this field is indeed still controversial and I strongly concur that further analysis is required to reduce vulnerability and uncertainty. In this respect, Reviewer 1's concerns are especially punctual.

We appreciate that the function of nuclear RTKs is a controversial field. A number of experiments we, and others, have carried out lend credence to the concept, that the spatial signalling of RTKs is not exclusive to the cell surface or endosomes, but can take place in the nucleus.

a) We have used a range of independent techniques: immunostaining, sub-cellular fractionation, pharmacological inhibition of FGFR1 and genetic inhibition of FGFR1 and FGF2, alongside observations from cell-cycle analysis, organotypic experiments as well *ex-vivo* analysis of human tissues, to demonstrate the functional relevance of nuclear FGFR1 and FGF2 in pancreatic stellate cells.
b) Efficient and reproducible FGFR1 and FGF2 RNAi, was able to abolish reciprocal nuclear FGF2 and FGFR1 localisation in pancreatic stellate cells.
c) This was consistent with other results, which suggest that the full length FGFR1 can localise to the nucleus (Somanathan et al, 2003). These observations have been supported by other studies that have shown nuclear FGFR1, using diverse antibodies and/or constructs with different epitope tags, when endogenous or recombinant FGFR1 was examined, respectively (Chioni & Grose, 2012; Peng et al, 2002a; Stachowiak et al, 1996; Stachowiak et al, 1997b). Finally, translocation of FGFR1 through the nuclear membrane and subsequent nuclear accumulation has also been demonstrated by immuno-electron microscopy (Stachowiak et al, 1996).

Thus the nuclear interior is a major sub-cellular site of functional FGFR1 and FGF2. In this manuscript we show the functional relevance of nuclear FGFR1 and FGF2 in pancreatic stellate cells for the first time.

Section B: Reviewer 1

We are very grateful for this reviewer's comments and suggestions. The clarification in a point-by-point manner is as follows:

1. *Fig. 1 and others: Nuclear localization data are not convincing? Preabsorbed antibody with FGFR1 or FGF2 are good negative controls, which will at least confirm that the staining are not just non-specific staining.*

We employ currently accepted 'gold standard' methods for determining nuclear localisation. Thus we demonstrate convincing, independent confocal z-stacking data (Figure 2A), co-localisation analysis (Figure 3B, Supporting Information Figure 1), but also sub-cellular fractionation and Western blotting (Figure 2F and 2M; Supporting Information Figure 5A).

Clearly, these methods all rely on antibody specificity. We have included multiple independent examples of Western blotting (as well as sub-cellular fractionation) and immunofluorescence after either FGFR1 or FGF2 RNAi in different cell lines (Figures 2 and 3 and Supporting Information Figures 4 and 8) to demonstrate specificity and sensitivity of the antibodies used. We also include controls with our Santa cruz anti-FGFR1 Western blotting antibody preabsorbed against the

immunising peptide (data not for publication: Related Manuscript File: Figure 1). As acknowledged by the reviewer, we have already provided thorough characterisation of Abcam FGFR1 antibody in a previous publication (Chioni & Grose, 2012).

2. *Figure 2, why FGF2 also go to nuclear independent of the receptor? What is the function of receptor-independent function of FGF2. If FGF2 or FGFR2 functions as a transcription factor, then chip-seq will be a good way to identify the responsive elements of the two molecules*

Indeed we observed diffuse and nucleolar FGF2 staining in the nucleus in addition to co-localisation with FGFR1 at nuclear speckles, shown by confocal z-stack in Figure 2A. The expanded discussion fully addresses the reviewer's comment.

In addition to FGF2 localising at speckles with FGFR1, we observed FGF2 in the nucleolus, a major site of ribosomal synthesis. FGF2 localisation to the nucleolus was first identified in adult bovine aortic cells, suggesting a role in driving quiescent cells into a proliferative state (Bouche et al, 1987). Nuclear localisation of FGF2 in these studies correlated with stimulation of transcription of ribosomal genes, during transition from G0 to G1 phase of the cell cycle, and increased expression of the major non-histone nucleolar protein, nucleolin, which has a key role in ribosomal transcription (Bugler et al, 1982). FGF2 also had a direct effect on the enhancement of RNA polymerase I activity in nuclear extracts isolated from quiescent cells, implying a mitogenic role for nuclear FGF2 (Joy et al, 1997; Peng et al, 2002b; Stachowiak et al, 1994). Therefore, it is possible that FGFR1 and a pool of FGF2 may influence distinct nuclear functional domains in PSCs. Studies have shown that FGFR1 interacts more with HMW FGF2 than LMW FGF2 (Dunham-Ems et al, 2009). Furthermore, HMW FGF2 can decrease the mobility of nuclear FGFR1 following stimulation, to facilitate the interaction of the receptor with gene promoters and other nuclear proteins such as CREB-binding protein (CBP), thus influencing transcriptional activity (Dunham-Ems et al, 2009). Thus, the nuclear localisation of FGF2 within distinct regions of the nuclei of PSCs may be driving distinct biological effects either with or without its receptor. The concept that nuclear FGFR1 and FGF2 may have a functional relationship exclusively in stellate cells was strengthened by the observation that, upon FGF2 knock-down, FGFR1 failed to translocate to the nucleus. However, no effect was observed on nuclear FGFR1 in cancer cells, which do not have nuclear FGF2.

We thank the reviewer for the suggestion to perform ChIP-seq to identify responsive elements of nuclear FGFR1. Indeed, since previous studies that suggest nuclear FGFR1 may associate with gene promoters (Peng et al, 2001a; Peng et al, 2002a; Stachowiak et al, 2003a), and given the speckled localisation of FGFR1 in stellate cells which has FGFR1 has previously shown to locate in neuronal cells (Somanathan et al, 2003), we performed preliminary ChIP experiments to identify the genomic regions with which FGFR1 may associate. This set of experiments proved challenging, given that much of what is known about nuclear FGFR1 is specific to neuronal cell gene regulation (Peng et al, 2002a; Stachowiak et al, 2003a) and those particular target genes are not expressed in pancreatic stellate cells, leaving us without a robust positive control. Therefore, we proceeded with hitherto uncharted ChIP-seq experiments using two antibodies (Abcam and Cell Signalling FGFR1) not validated for this purpose. We used Illumina ChIP-Seq (Mi-Seq). This was outsourced to a company (Active Motif). The initial analysis done by Active Motif was received by us two weeks ago. It yielded poor enrichment (Data not for publication Related Manuscript File Figure 2). This raises two possibilities, either the antibodies used were not suitable for ChIP-seq experiments, a well recognised problem in the field (Bao et al, 2013; Park, 2009), or, possibly, the role of the receptor in the nucleus may not be to directly associate with chromatin but may be to function in another manner to regulate cell behaviour such as acting as a scaffold to phosphorylate other proteins in the nucleus (Hu et al, 2004).

We currently are trying to rectify these technical difficulties by

1. Re-analysing the large dataset with in-house bio-informatics.
2. Screening a panel of FGFR1 antibodies to identify a candidate that may be taken forward

as a robust antibody for future ChIP-seq experiments

3. Generating inducible epitope-tagged constructs that will allow us to circumvent these problems in the future.

Thus, we demonstrate our commitment to addressing the reviewer's valuable suggestion, which we have taken forward in a step-wise, logical manner. As you will appreciate, the technical limitations prevent us from providing Chip-seq data in the given time frame, despite our best efforts to conduct these experiments.

The corroborative evidence of the functional role of nuclear FGFR1 and other RTKs in regulating gene expression in other cell types (Chioni & Grose, 2012; Hung et al, 2008; Lin et al, 2001; Lo et al, 2005; Peng et al, 2001a; Peng et al, 2002a; Somanathan et al, 2003; Stachowiak et al, 2003a; Wang et al, 2004) is overwhelming.

3. *Fig. 2E, fgf2 treatment should be done to see it exogenous FGF2 can induce FGFR1 nuclear localization.*

We thank reviewer for this suggestion, which strengthens our dataset supporting our initial hypothesis that FGF2 can induce nuclear localisation of FGR1. We now show that treatment of PSCs with recombinant FGF2 induces rapid nuclear accumulation of both FGFR1 and FGF2 (Supporting Information Figure 6 G-I).

4. *Fig. 2E need to show that the activity of FGFR1 is specific, other three non-FGFR1 expressing cells should be used as a control, the same is needed for Fig. 5*

This has been a valuable suggestion to demonstrate specificity of FGFR1. We show decreased nuclear FGFR1 upon FGF2 knockdown in PSCs (improved Figure 2 C-I and Supporting Information Figure 4). We also show that culturing PSCs treated with FGFR1 RNAi in our organoytpic model abolishes cell invasion, demonstrating the activity of FGFR1 is specific (new Figure 6E,F).

5. *Fig. 2J, why only some cells are inhibited? Different dosages should be used to show the activity is dosage dependent*

We demonstrate a dose dependent effect of PD173074 on nuclear FGFR1 in PSCs (new data in Supporting Information 5B). The effect of nuclear FGFR1 on cell cycle (improved Figure 4) may explain why all cells are not inhibited, and this is an area of current research in our Lab.

6. *Fig. 3A, B, why fgf2 does not colocalized with sc35? Does it bind to fgfr1?*

We have shown that FGFR1 and FGF2 co-localise at speckles in PSCs (Figure 2A, Supporting Information Figure 3B) but not cancer cells (Supporting Information Figure 2). Technical limitations (both SC35 and FGF2 antibodies being raised in the same species) prevent us from demonstrating FGF2 co-localisation to SC35 domains (Figure 3A).

7. *Fig. 6 ac, why fgfr1 is punctate stained and fgf2 is diffusely stained. Do they colocalized?*

© EMBO

It is correct that there is a diffuse staining as well as a speckled pattern of FGF2 (Figure 2 and others) in PSC and other cell lines. This is addressed in the discussion (see Section B, Comment 2,).

8. *Fig. 8, need high resolution and high magnification pictures for evaluation*

We now include magnified high resolution images, in improved Figure 8, which clearly demonstrate nuclear FGFR1 and FGF2 at the invasive front.

Section C: Reviewer 2

We are pleased to note that the reviewer finds our experiments to be well designed and important in advancing the field. We have addressed points as follows:

1. *Authors did not touch the interesting matter concerning the downstream signaling of nuclear FGFR1. They just restricted their comments that the nuclear receptor-induced effects in PS1 cells are sensitive to PD173074 treatment or FGFR1 depletion and that a treatment with the inhibitor resulted also in a significant reduction in nuclearFGF2 (both isoform) as well as full length FGFR1. The lack of a piece of rational speculations on this makes the story more difficult to "digest".*

We have provided detailed arguments as to the potential downstream effects of nuclear FGFR1 and FGF2 (see excerpts from Discussion below). As discussed above (Section B, Comment 2), we used a ChIP-seq approach to try and drill down further into potential nuclear function, but have been hampered by technical difficulties.

"Confocal analysis revealed co-localisation of FGFR1 and FGF2 at distinct nuclear speckles (Peng et al, 2002b; Stachowiak et al, 2003b), sites of RNA polymerase II mediated transcription and co-transcriptional pre-mRNA processing (Crispino et al, 1994), suggesting that nuclear FGFR1 and FGF2 regulate transcription of genes involved in proliferation. Indeed, FGFR1 can bind effectively to all isoforms of FGF2; the LMW isoform, which is generally extracellular, as well as the predominantly nuclear HMW isoforms. Studies have shown that HMW FGF2 may be required to regulate the nuclear entry and mobilisation of FGFR1, facilitating the interaction between FGFR1 and gene promoters and other nuclear proteins, thereby regulating transcription (Dunham-Ems et al, 2009).

In addition to FGF2 localising at speckles with FGFR1, we observed FGF2 in the nucleolus, a major site of ribosomal synthesis. FGF2 localisation to the nucleolus was first identified in adult bovine aortic cells, suggesting a role in driving quiescent cells into a proliferative state (Bouche et al, 1987). Nuclear localisation of FGF2 in these studies correlated with stimulation of transcription of ribosomal genes, during transition from G0 to G1 phase of the cell cycle, and increased expression of the major non-histone nucleolar protein, nucleolin, which has a key role in ribosomal transcription (Bugler et al, 1982). FGF2 also had a direct effect on the enhancement of RNA polymerase I activity in nuclear extracts isolated from quiescent cells, implying a mitogenic role for nuclear FGF2 (Joy et al, 1997; Peng et al, 2002b; Stachowiak et al, 1994). Therefore, it is possible that FGFR1 and a pool of FGF2 may influence distinct nuclear functional domains in PSCs. Studies have shown that FGFR1 interacts more with HMW FGF2 than LMW FGF2 (Dunham-Ems et al, 2009). Furthermore, HMW FGF2 can decrease the mobility of nuclear FGFR1 following stimulation, to facilitate the interaction of the receptor with gene promoters and other nuclear proteins such as CREB-binding protein (CBP), thus influencing transcriptional activity (Dunham-Ems et al, 2009). Thus, the nuclear localisation of FGF2 within distinct regions of the nuclei of PSCs may be driving distinct biological effects either with or without its receptor. …..

Silencing FGF2 or FGFR1, or blocking FGFR signalling with PD173074, in PSCs resulted in significant reduction in cell proliferation. These effects were not apparent in PDAC cell lines, which did not display nuclear FGF2, suggesting that nuclear FGFR1 and FGF2 may co-activate genes involved in cellular proliferation exclusively in PSCs. Indeed, nuclear FGFR1 has been shown to regulate *c-Jun* and *Cyclin D1* in human glial cells (Reilly & Maher, 2001). Blocking nuclear FGFR1 and FGF2 in PSCs, using PD173074, correlated with a G1 cell-cycle block and a significant reduction in cyclin D1 expression. Activation of cyclin D1 by nuclear FGFR1 and FGF2 may drive entry into the cell cycle, as has been shown in neuronal cells (Joy et al, 1997; Stachowiak et al, 1997a). Moreover, nuclear FGFR1 has been shown to regulate *FGF2* gene expression by indirectly activating the *FGF2* promoter (via cAMP and PKC dependent signalling pathways) (Peng et al, 2001b). We also observed a significant reduction in FGF2 expression following FGFR1 knock-down exclusively in PSCs, with no effect in PDAC cells, providing further evidence for the critical role of nuclear FGFR1-driven proliferation in PSCs."

2. *I am curious if FGF2/FGFR1 will still be translocated to the nuclear compartment under FRS2 depletion?*

We thank the reviewer for this valuable insight. We now include new data in Supporting Information Figure 6 A-F demonstrating depletion of FRS2 has a profound effect on nuclear FGFR1 and FGF2.

Section D. Reviewer 3.

We are pleased to note that the reviewer finds our experiments to be an interesting and that the study may advance the field. We have addressed several points as follows.

1. *In figure 1, the relationship between cells counted and the correlation within each patient is not clear. In Figure 1B and 1D, What does n=46 refer to? Number of cells from each patient examined? In figure 1F there appears to be 36 data points, was this all patients on the TMA?*

We analysed 46 patients. Of those 46 patients, 36 were successfully scored for both FGFR1 and FGF2 and we could analyse the relationship between nuclear FGFR1 and FGF2 in the stromal fibroblasts in these patients. We have now updated Figure 1 B and D to include 'n=46', furthermore we have updated the Figure Legend and Methods section to make this clearer.

2. *The graph in Figure 2A was a little confusing, would the X-axis be better labelled as colocalization rather than PS1. In Figure 2D is each data point 1 cell (presumably not) or the average of a replicate experiment including multiple cells/images. For figures 2H-L, there are12 data points but no explanation as to what each data point is summarizing, one image or the average of multiple images from one replicate experiment? Please add explanation to methods.*

   We apologise for the lack of clarity and have now changed the x-axis in the improved Figure 2 A to 'FGFR1 and FGF2 co-localisation' in order to address the reviewer's comment. In updated Figure 2D, E and 2H-L, each data point shown represents an average of PS1 total or nuclear FGFR or FGF2 per field. Several fields were counted per experiment. For all experimental conditions, images are representative of three independent experiments the total number of PS1 cells analysed is now recorded in the figure (n) and we have updated Methods section and Figure Legends to make this clearer.

'Multiple fields (at least 3) were taken per experiment and the total cells per field were analysed. An average of total, nuclear FGFR or FGF2 per field was plotted. All experiments were carried out on three separate occasions. The total number of cells analysed is recorded in each figure.'

3. *For Figure 3C-H and 4B, the authors graph % Ki67 cells but do not state how many cells were examined to get this percentage? In figure 6C and 7C, the authors graph % of PS cells with nuclear FGFR1/FGF2, again how many cells were analysed in each replicate experiment to obtain percentage. Similar issue with Figure 8C.*

We have now added the total number of cells that were analysed in each experiment to the restructured Figure 3C-H, 4B, 6C, 7C and 8E and H. We have also updated the figure legends for these images and Methods section to make this clearer.

'Multiple fields (at least 3) were taken per experiment and the total cells per field were analysed. An average of total, nuclear FGFR or FGF2 per field was plotted. All experiments were carried out on three separate occasions. The total number of cells analysed is recorded in each figure'

'Ki67 staining was also used to identify proliferating cells. Each data point plotted represents the percentage of cells positive for Ki67 per field. Multiple fields were taken per experiment. The total number of cells analysed to obtain this percentage was recorded in the figure.'

4. *In figure 5D, can the authors add how many HPF were counted in each replicate experiment to the methods?*

We analysed twelve HPF per organotypic gel and have now updated Figure 5D to show the average number of invading cohorts per HPF, which is shown by each data point. Nine gels were analysed in total from three separate experiments. In addition we have updated the Figure legend and Methods section to make this clearer

'To analyse the percentage of cells with nuclear FGFR1 and FGF2 in organotypic sections, multiple fields per gel were analysed. The average percentage of PS1 cells across these fields with nuclear FGFR1 or FGF2 were plotted and are shown by each graphical data point. Nine gels were analysed in total from three separate experiments. In order to analyse the percentage of nuclear FGFR1 and FGF2 positive fibroblasts at the invasive front or centre of the tumour in tissue sections, several fields per section per patient (four patients) were analysed. Each data point is representative of one HPF. The total number of fibroblasts analysed for all the patients is recorded in the figure'.

5. *In relation to the organotypic assays, figure 7 is convincing. Stellate cell nuclear FGF2 localisation is very clear in the control, as is the failure to localise in the nucleus, in the presence of inhibitor. However, figure 6, is not convincing. FGFR1 looks to be no different in control and treatment conditions in the vimentin positive-stellate cells. This may be due to the choice of representative image. Alternatively, given the importance of the organotypic model system employed in validating the proposed mechanism, the paper would be strengthened by digital quantitation of the nuclear FGFR1 in invasive cells versus "trapped PSCs" following PD173074. For example, could authors apply thresholding to quantify nuclear FGFR2 in the vimementin positive cells.*

© EMBO

We provide better images in Figure 6 A and B to show the difference in nuclear FGFR1 in invasive compared to trapped PS1 cells, and provide automated digital quantification in the reorganised Figure 6 D.

6. *In the organotypic culture assays, the authors only use the inhibitor which does not differentiate between FGF/FGFR family members. The manuscript would be strengthened by depleting stellate cells of FGFR1 only and show that invasion does not occur in these organotypic assays.Addressing comments 1-4 are essential, addressing comment 5 is highly desirable.*

We have now included Figure 6 E-F, which shows that culturing stellate cells that have been treated with FGFR1 RNAi, in our mini-organotypic model, leads to a significant decrease in cell invasion.

Minor Issues:

Figure legends were a little confusing, often grouping figures A-D, todescribe something. The authors should consider expanding the figure legends to describe each item in the figure.

We have now expanded the Figure legends to describe the Figures in more detail.

References

Bao Y, Vinciotti V, Wit E, t Hoen PA (2013) Accounting for immunoprecipitation efficiencies in the statistical analysis of ChIP-seq data. *BMC Bioinformatics* 14: 169

Bouche G, Gas N, Prats H, Baldin V, Tauber JP, Teissie J, Amalric F (1987) Basic fibroblast growth factor enters the nucleolus and stimulates the transcription of ribosomal genes in ABAE cells undergoing G0----G1 transition. *Proceedings of the National Academy of Sciences of the United States of America* 84: 6770-6774

Bugler B, Caizergues-Ferrer M, Bouche G, Bourbon H, Amalric F (1982) Detection and localization of a class of proteins immunologically related to a 100-kDa nucleolar protein. *Eur J Biochem* 128: 475-480

Chioni AM, Grose R (2012) FGFR1 cleavage and nuclear translocation regulates breast cancer cell behavior. *The Journal of cell biology* 197: 801-817

Crispino JD, Blencowe BJ, Sharp PA (1994) Complementation by SR proteins of pre-mRNA splicing reactions depleted of U1 snRNP. *Science* 265: 1866-1869

Dunham-Ems SM, Lee YW, Stachowiak EK, Pudavar H, Claus P, Prasad PN, Stachowiak MK (2009) Fibroblast growth factor receptor-1 (FGFR1) nuclear dynamics reveal a novel mechanism in transcription control. *Molecular biology of the cell* 20: 2401-2412

Hu Y, Fang X, Dunham SM, Prada C, Stachowiak EK, Stachowiak MK (2004) 90-kDa ribosomal S6 kinase is a direct target for the nuclear fibroblast growth factor receptor 1 (FGFR1): role in FGFR1 signaling. *J Biol Chem* 279: 29325-29335

Hung LY, Tseng JT, Lee YC, Xia W, Wang YN, Wu ML, Chuang YH, Lai CH, Chang WC (2008) Nuclear epidermal growth factor receptor (EGFR) interacts with signal transducer and activator of transcription 5 (STAT5) in activating Aurora-A gene expression. *Nucleic Acids Res* 36: 4337-4351

Joy A, Moffett J, Neary K, Mordechai E, Stachowiak EK, Coons S, Rankin-Shapiro J, Florkiewicz RZ, Stachowiak MK (1997) Nuclear accumulation of FGF-2 is associated with proliferation of human astrocytes and glioma cells. *Oncogene* 14: 171-183

Lin SY, Makino K, Xia W, Matin A, Wen Y, Kwong KY, Bourguignon L, Hung MC (2001) Nuclear localization of EGF receptor and its potential new role as a transcription factor. *Nat Cell Biol* 3: 802-808

Lo HW, Hsu SC, Ali-Seyed M, Gunduz M, Xia W, Wei Y, Bartholomeusz G, Shih JY, Hung MC (2005) Nuclear interaction of EGFR and STAT3 in the activation of the iNOS/NO pathway. *Cancer Cell* 7: 575-589

Park PJ (2009) ChIP-seq: advantages and challenges of a maturing technology. *Nat Rev Genet* 10: 669-680

Peng H, Moffett J, Myers J, Fang X, Stachowiak EK, Maher P, Kratz E, Hines J, Fluharty SJ, Mizukoshi E, Bloom DC, Stachowiak MK (2001a) Novel nuclear signaling pathway mediates activation of fibroblast growth factor-2 gene by type 1 and type 2 angiotensin II receptors. *Molecular biology of the cell* 12: 449-462

Peng H, Moffett J, Myers J, Fang X, Stachowiak EK, Maher P, Kratz E, Hines J, Fluharty SJ, Mizukoshi E, Bloom DC, Stachowiak MK (2001b) Novel nuclear signaling pathway mediates activation of fibroblast growth factor-2 gene by type 1 and type 2 angiotensin II receptors. *Mol Biol Cell* 12: 449-462

Peng H, Myers J, Fang X, Stachowiak EK, Maher PA, Martins GG, Popescu G, Berezney R, Stachowiak MK (2002a) Integrative nuclear FGFR1 signaling (INFS) pathway mediates activation of the tyrosine hydroxylase gene by angiotensin II, depolarization and protein kinase C. *Journal of neurochemistry* 81: 506-524

Peng H, Myers J, Fang X, Stachowiak EK, Maher PA, Martins GG, Popescu G, Berezney R, Stachowiak MK (2002b) Integrative nuclear FGFR1 signaling (INFS) pathway mediates activation of the tyrosine hydroxylase gene by angiotensin II, depolarization and protein kinase C. *J Neurochem* 81: 506-524

Reilly JF, Maher PA (2001) Importin beta-mediated nuclear import of fibroblast growth factor receptor: role in cell proliferation. *The Journal of cell biology* 152: 1307-1312

Somanathan S, Stachowiak EK, Siegel AJ, Stachowiak MK, Berezney R (2003) Nuclear matrix bound fibroblast growth factor receptor is associated with splicing factor rich and transcriptionally active nuclear speckles. *J Cell Biochem* 90: 856-869

Stachowiak EK, Fang X, Myers J, Dunham S, Stachowiak MK (2003a) cAMP-induced differentiation of human neuronal progenitor cells is mediated by nuclear fibroblast growth factor receptor-1 (FGFR1). *Journal of neurochemistry* 84: 1296-1312

Stachowiak EK, Fang X, Myers J, Dunham S, Stachowiak MK (2003b) cAMP-induced differentiation of human neuronal progenitor cells is mediated by nuclear fibroblast growth factor receptor-1 (FGFR1). *J Neurochem* 84: 1296-1312

Stachowiak EK, Maher PA, Tucholski J, Mordechai E, Joy A, Moffett J, Coons S, Stachowiak MK (1997a) Nuclear accumulation of fibroblast growth factor receptors in human glial cells--association with cell proliferation. *Oncogene* 14: 2201-2211

Stachowiak MK, Maher PA, Joy A, Mordechai E, Stachowiak EK (1996) Nuclear accumulation of fibroblast growth factor receptors is regulated by multiple signals in adrenal medullary cells. *Molecular biology of the cell* 7: 1299-1317

Stachowiak MK, Moffett J, Joy A, Puchacz E, Florkiewicz R, Stachowiak EK (1994) Regulation of bFGF gene expression and subcellular distribution of bFGF protein in adrenal medullary cells. *The Journal of cell biology* 127: 203-223

Stachowiak MK, Moffett J, Maher P, Tucholski J, Stachowiak EK (1997b) Growth factor regulation of cell growth and proliferation in the nervous system. A new intracrine nuclear mechanism. *Mol Neurobiol* 15: 257-283

Wang SC, Lien HC, Xia W, Chen IF, Lo HW, Wang Z, Ali-Seyed M, Lee DF, Bartholomeusz G, Ou-Yang F, Giri DK, Hung MC (2004) Binding at and transactivation of the COX-2 promoter by nuclear tyrosine kinase receptor ErbB-2. *Cancer Cell* 6: 251-261

2nd Editorial Decision 05 December 2013

Thank you for the submission of your revised manuscript to EMBO Molecular Medicine. We have now received the enclosed reports from the referees that were asked to re-assess it. As you will see the reviewers are now globally supportive and I am pleased to inform you that we will be able to accept your manuscript pending the following final amendments:

1) As per our Author Guidelines, the description of all reported data that includes statistical testing must state the name of the statistical test used to generate error bars and P values, the number (n) of independent experiments underlying each data point (not replicate measures of one sample), and the actual P value for each test (not merely 'significant' or 'P < 0.05').

2) We would need a short list (up to 5) of bullet points that summarize the key NEW findings. The bullet points should be designed to be complementary to the abstract and will be used online in our new platform (coming January 2014).

3) We are now encouraging the publication of source data, particularly for electrophoretic gels and blots, with the aim of making primary data more accessible and transparent to the reader. Would you be willing to provide a PDF file per figure that contains the original, uncropped and unprocessed scans of all or at least the key gels used in the manuscript? The PDF files should be labeled with the appropriate figure/panel number, and should have molecular weight markers; further annotation may be useful but is not essential. The PDF files will be published online with the article as supplementary "Source Data" files. If you have any questions regarding this just contact me.

Please submit your revised manuscript within two weeks. I look forward to seeing a revised form of

your manuscript as soon as possible.

***** Reviewer's comments *****

Referee #2

None

Referee #3 (Remarks):

The authors have responded to my individual critiques. They have expanded the figure legends and added essential details to the methods and figure legends as to the number of biological replicates (n=3), the number of fields examined and or total number of cells examined that significantly strengthens the paper. They have also digitally quantified their immunofluorescent images as requested. The authors have outlined their responses to the reviewers criticisms in great detail and should be commended.

2nd Revision - authors' response 17 December 2013

Please find attached the final revisions to our manuscript "Nuclear translocation of FGFR1 and FGF2 in pancreatic stellate cells facilitates pancreatic cancer cell invasion", that has been accepted for publication in EMBO Molecular Medicine. We have put the full P values, as requested and uploaded source data files for western blots.

Many thanks for your help in handling our manuscript. The review process has added significant strengths to the paper and we are extremely grateful for all the positive inputs that we have received.
