# Supplementary figures and images for: Nuclear translocation of FGFR1 and FGF2 in pancreatic stellate cells facilitates pancreatic cancer cell invasion
Source: EMBO Mol Med. 2014 Feb 6;6(4):467–81. doi: 10.1002/emmm.201302698 (PMC3992074; doi:10.1002/emmm.201302698)

Supporting Information Fig 1

A

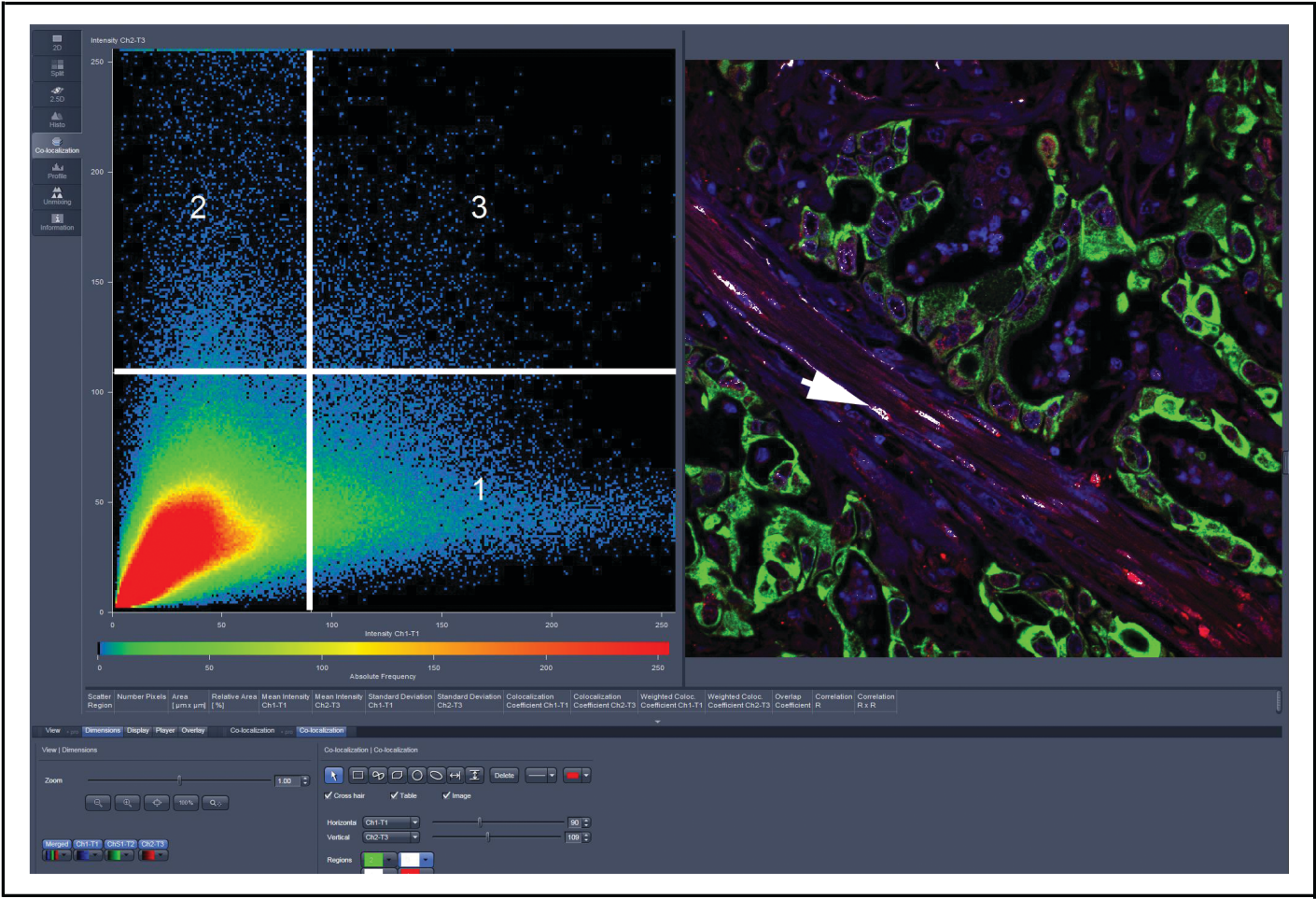

B

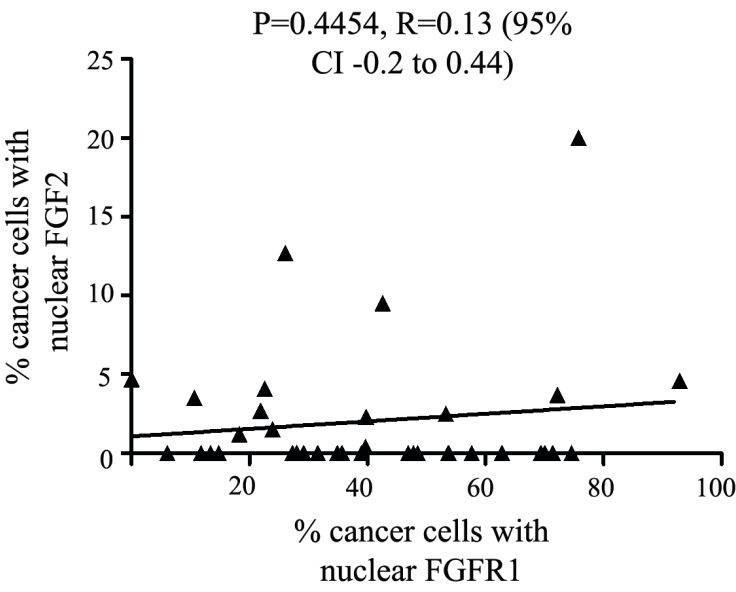

Supplement: Supplementary file 2 [file emmm0006-0467-sd2.pdf]

A

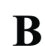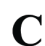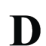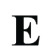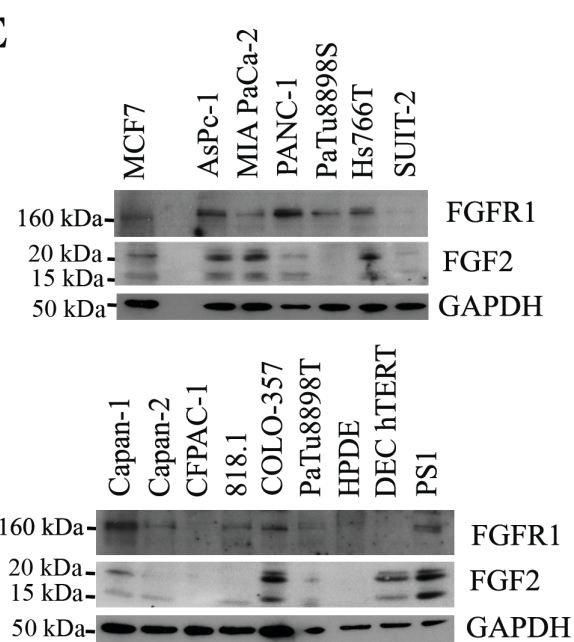

Supplement: Supplementary file 3 [file emmm0006-0467-sd3.pdf]

Supporting Information Fig 3

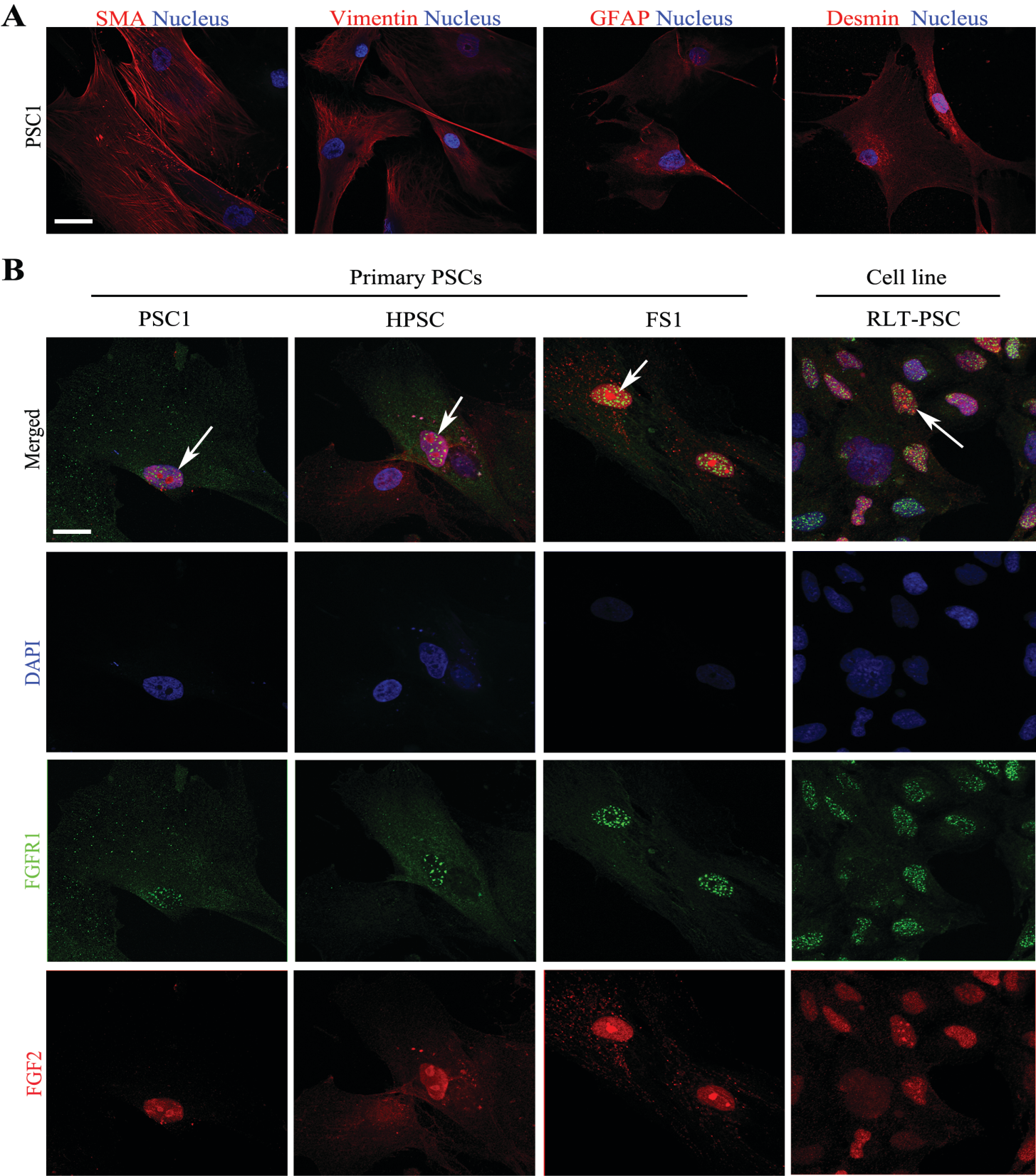

Supplement: Supplementary file 4 [file emmm0006-0467-sd4.pdf]

Supporting Information Fig 4

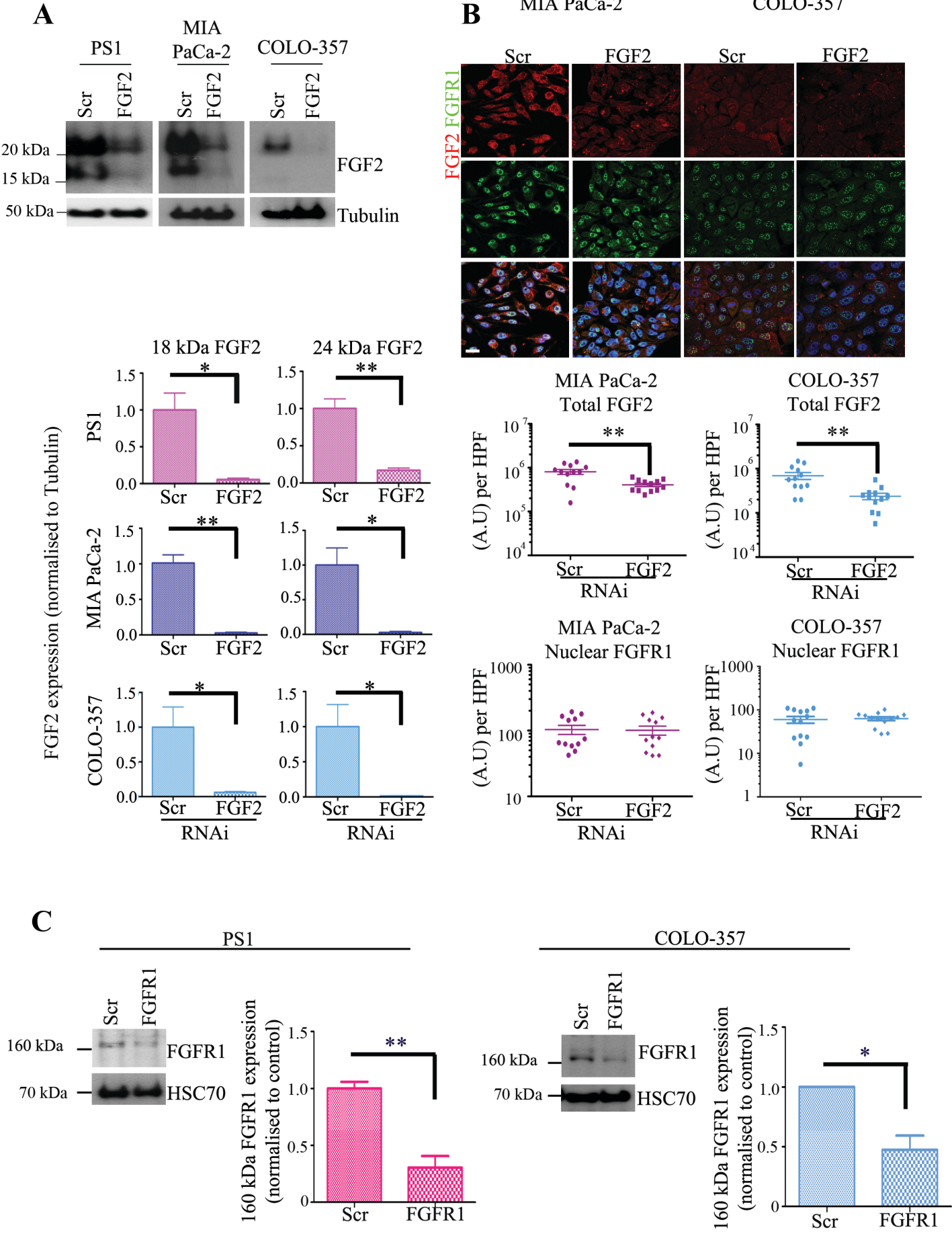

Supplement: Supplementary file 5 [file emmm0006-0467-sd5.pdf]

A

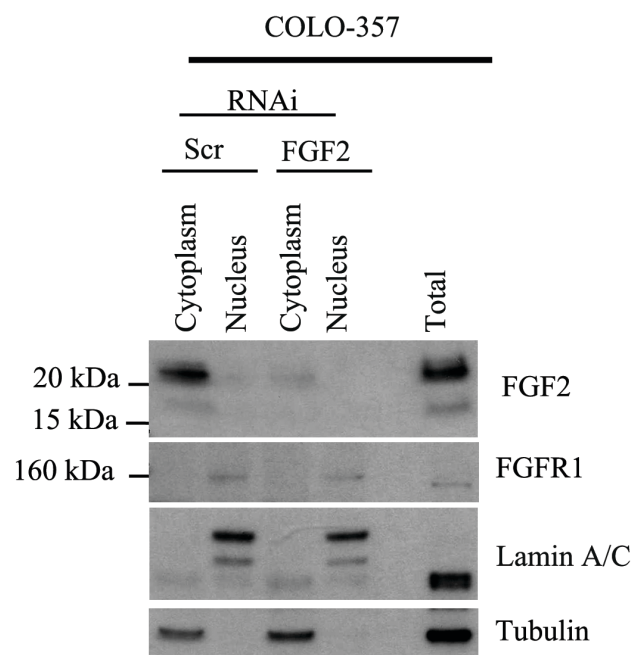

# B

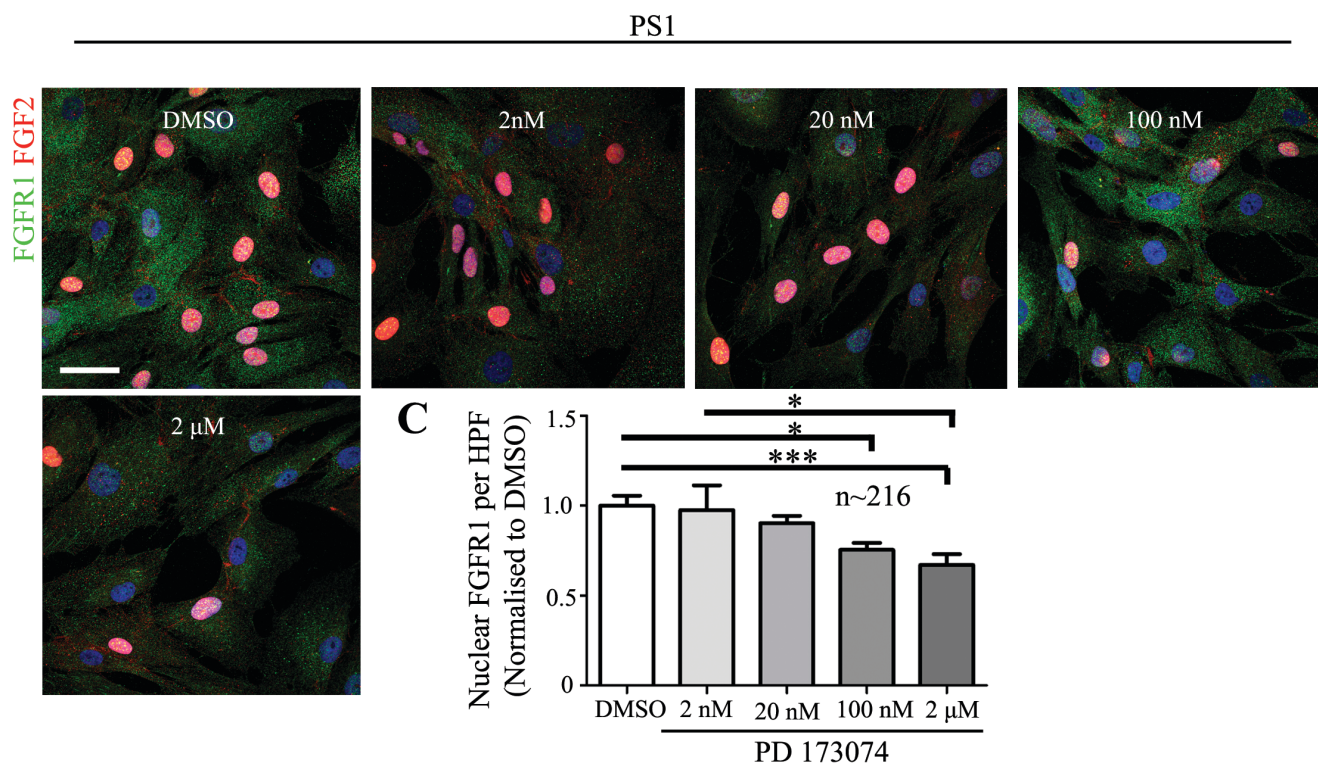

D

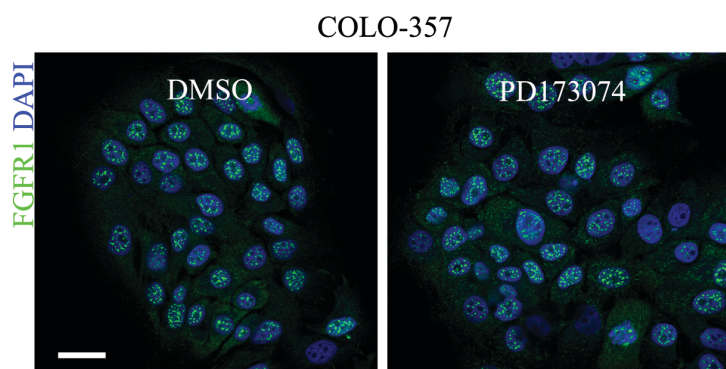

**E**

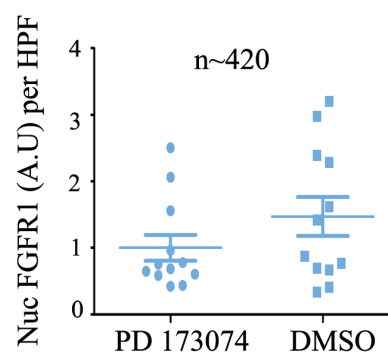

Supplement: Supplementary file 6 [file emmm0006-0467-sd6.pdf]

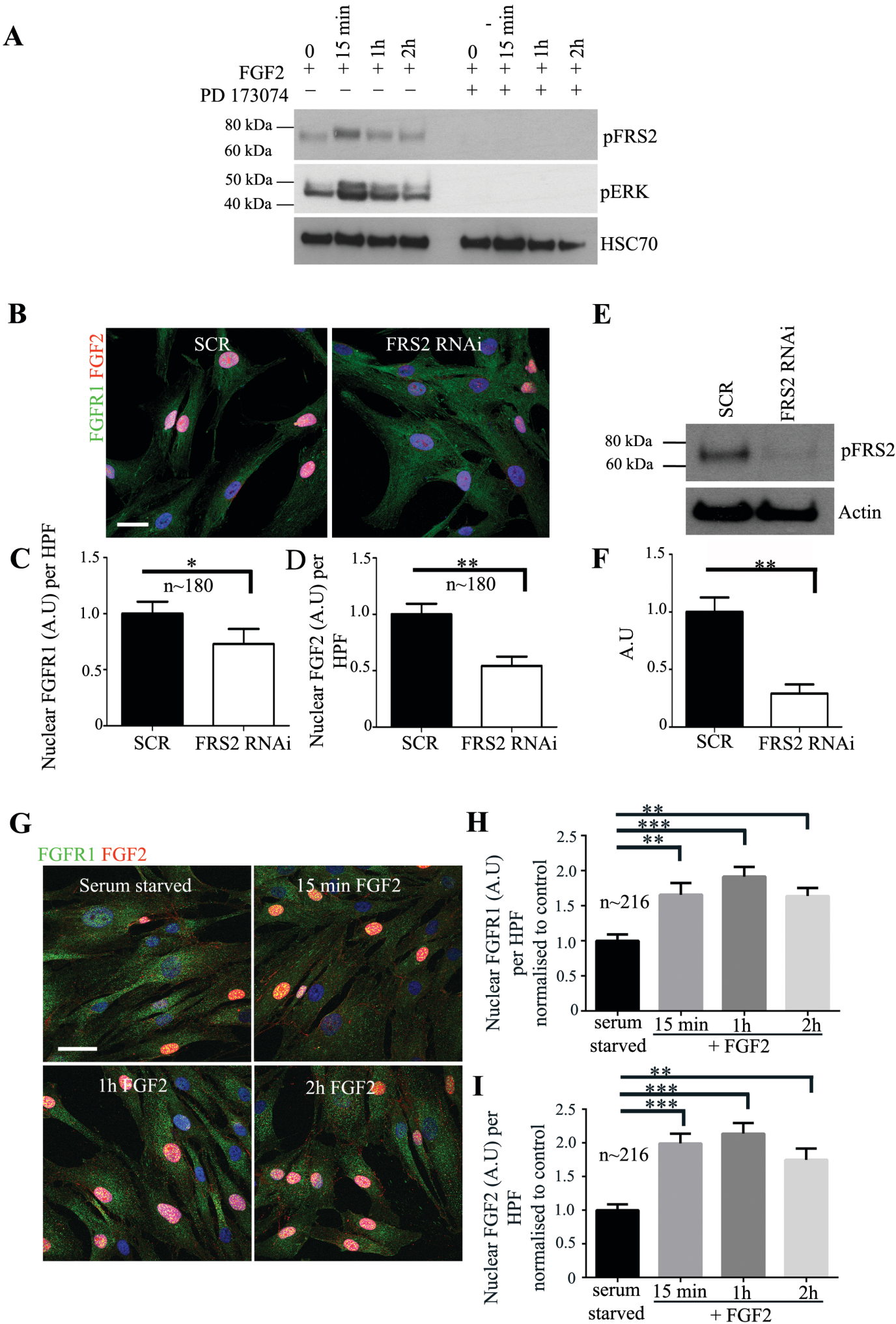

Supplement: Supplementary file 7 [file emmm0006-0467-sd7.pdf]

Supporting Information Fig 7

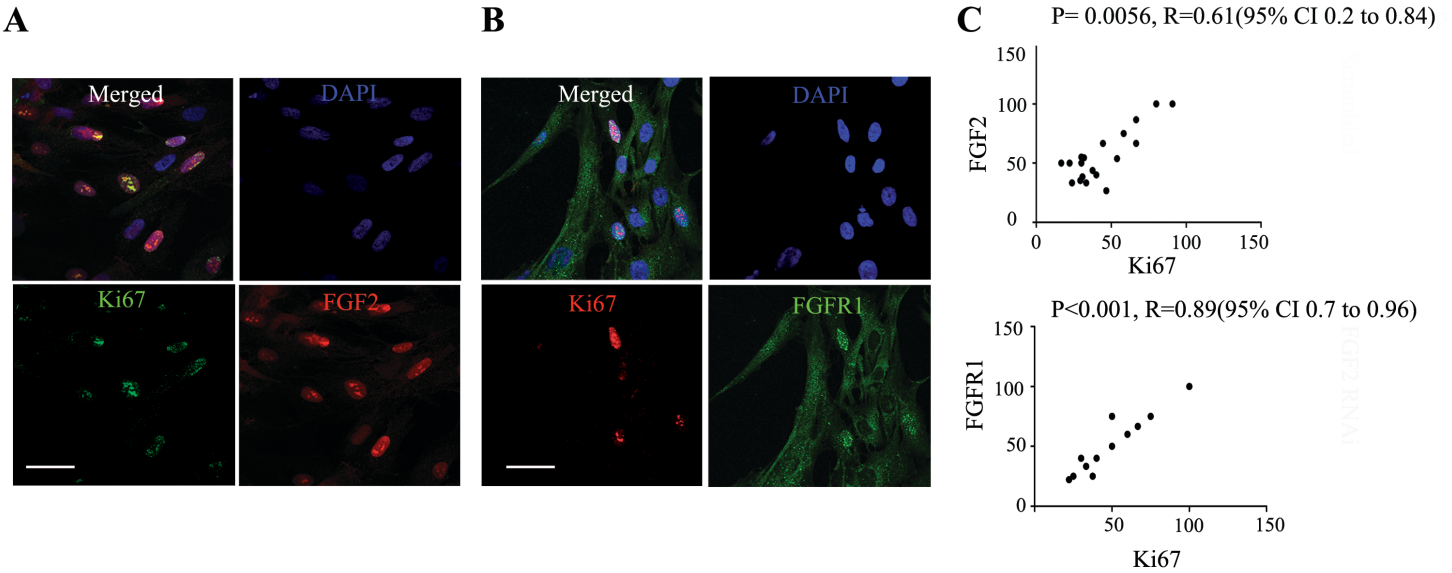

Supplement: Supplementary file 8 [file emmm0006-0467-sd8.pdf]

Supporting Information Fig 8

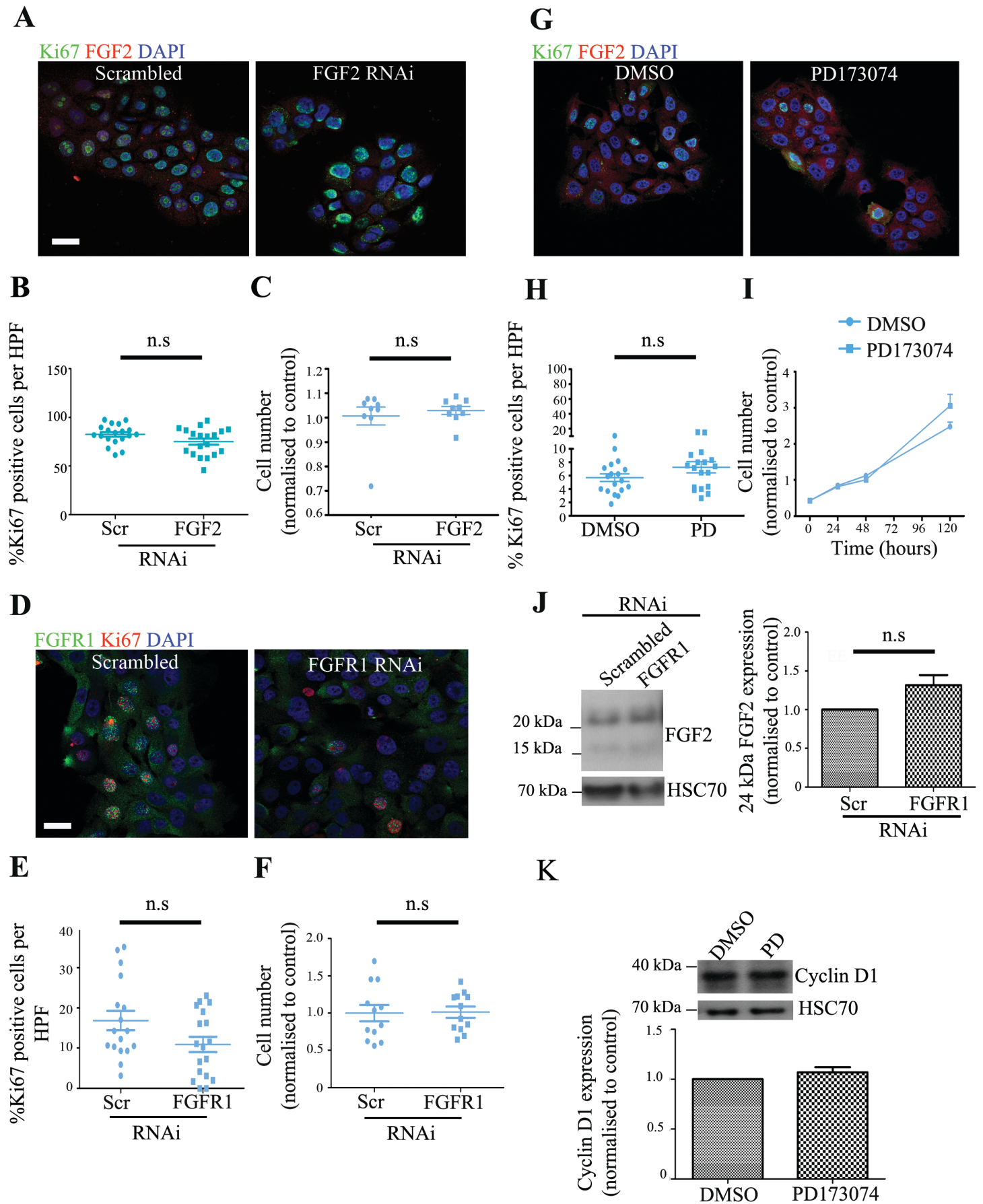

Supplement: Supplementary file 9 [file emmm0006-0467-sd9.pdf]

Supporting Information Fig 9

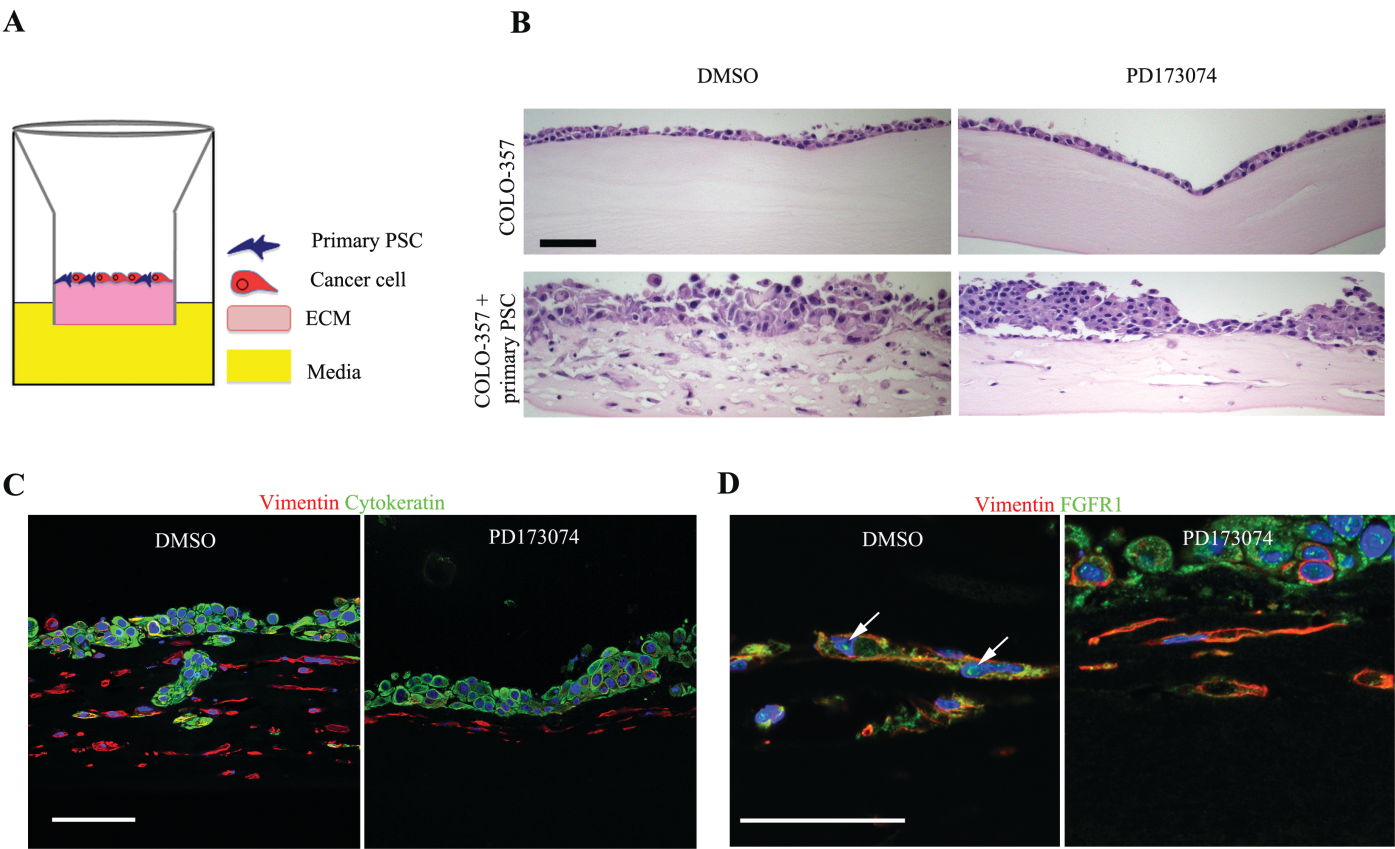

Supplement: Supplementary file 10 [file emmm0006-0467-sd10.pdf]

Supporting Information Fig 10

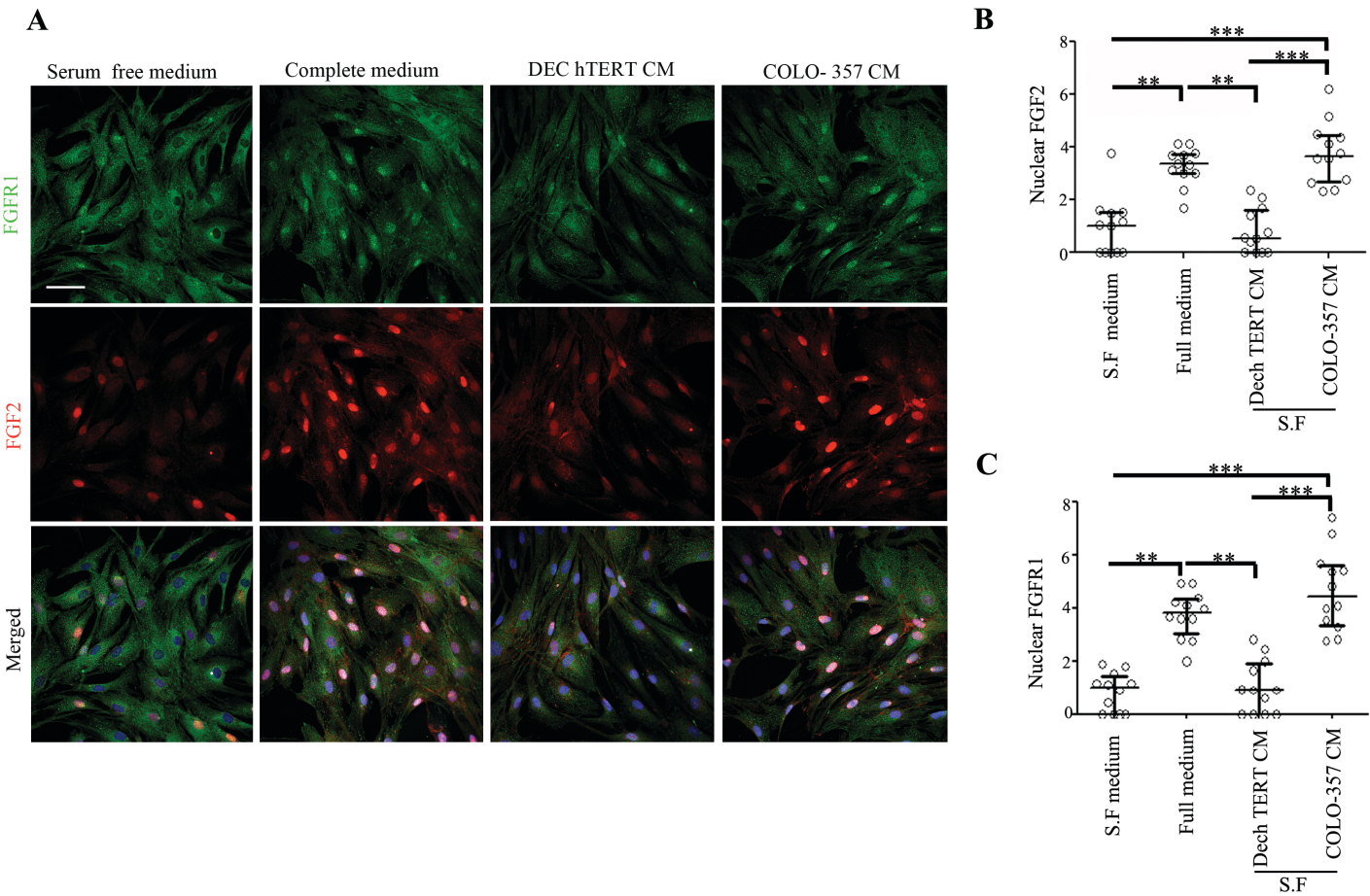

Supplement: Supplementary file 11 [file emmm0006-0467-sd11.pdf]
