# Supplementary material for: Nuclear translocation of FGFR1 and FGF2 in pancreatic stellate cells facilitates pancreatic cancer cell invasion
Source: EMBO Mol Med. 2014 Feb 6;6(4):467–81. doi: 10.1002/emmm.201302698 (PMC3992074; doi:10.1002/emmm.201302698)
Supplement: Supplementary file 12 [file emmm0006-0467-sd12.pdf]

Supporting Information Table 1

Cell numbers

Experiment	Total cell numbers	Cancer cells	Stellate cells (PS1 unless specified)
Cancer cells alone organotypic		5×10^5	0
Cancer: stellate cells organotypic	5×10^5	1.66×10^5	3.33×10^5
Cancer cells alone mini- organotypic		1×10^5	0
Cancer: stellate cells mini- organotypic	1×10^5	0.33×10^5	0.66×10^5 (primary PSC)
FGF stimulation assays			1×10^5
Cell growth assays			5×10^4
FACS (PI)			2.5×10^5
