# Supplementary material for: Nuclear translocation of FGFR1 and FGF2 in pancreatic stellate cells facilitates pancreatic cancer cell invasion
Source: EMBO Mol Med. 2014 Feb 6;6(4):467–81. doi: 10.1002/emmm.201302698 (PMC3992074; doi:10.1002/emmm.201302698)
Supplement: Supplementary file 13 [file emmm0006-0467-sd13.pdf]

Supporting Information Table 2

Antibodies

All antibodies are summarised in Table 2

Antibody	Species raised in	Supplier (Cat No)	Dilution for IF or IHC	Dilution for WB
HSC70	Mouse	Santa Cruz (SC7298)	N.A.	1:1000
αSMA	Rabbit	Abcam (ab5694)	1:100	NA
αSMA	Mouse	Dako clone 1A4 (M0851)	1:300	1:100
Vimentin	Mouse	Dako clone V9 (M0725)	c.s 1:2000<br>FFPE 1:50	1:250
Ki67	Rabbit	Abcam (ab15580)	c.s 1:100<br>FFPE 1:100	N.A
FGFR1 (recognises intracellular epitope in C terminus)	Rabbit	Santa Cruz (SC121)	N.A	1:1000
FGFR1 (recognises extracellular epitope near transmembrane domain)	Rabbit	Abcam (10646)	c.s 1:100<br>FFPE 1:500	N.A
Tubulin	Mouse	Sigma (T5168)	N.A	1:2000
Lamin A/C	Goat	Santa Cruz (SC6215)	N.A	1.1000
Actin	Goat	Santa Cruz (SC1615)		
FGF2	Rabbit	Peprotech (500-P18)	N.A	1:500
FGF2	Mouse	Millipore (05-118)	c.s 1:100<br>FFPE 1:100	N.A
pFRS2	Rabbit	Cell Signalling (38645)	N.A	1:1000
pERK	Rabbit	Cell Signalling (91015)	N.A	1:1000
SC35	Mouse	Sigma (S4045)	1:1000	NA
Cyclin D1	Mouse	Cell Signalling (2926P)	N.A	1:1000
Cytokeratin WSS	Rabbit	Dako (Z0622)	1:500	N.A
Anti-mouse HRP	Goat	Dako (P0447)	N.A	1:1000

**Antibody**	**Species raised in**	**Supplier (Cat No)**	**Dilution for IF or IHC**	**Dilution for WB**
HSC70	Mouse	Santa Cruz (SC7298)	N.A.	1:1000
αSMA	Rabbit	Abcam (ab5694)	1:100	NA
Anti-mouse HRP	Rabbit	Dako (P0260)	N.A	1:1000
Anti-goat HRP	Rabbit	Dako (P0160)	N.A	1:1000
Fluorescein-goat–anti rabbit	Rabbit	Invitrogen (F27 65)	1:100	N.A
Cy3 donkey anti-mouse	Mouse	Jackson Immunoresearch (715-166-151)	1:100	N.A

HSC70, heat shock cognate 70 kDa protein 8; αSMA, α-smooth muscle actin; Cytokeratin WSS, wide spectrum screening, recognising a broad spectrum of human cytokeratin; FGFR1, Fibroblast growth factor receptor 1; FGF2, Fibroblast growth factor 2; HRP, Horseradish peroxidase; c.s, coverslip; FFPE, formalin fixed paraffin embedded; IF, immunofluorescence; IHC, immunohistochemistry; WB, Western blot; N.A: not applicable.
