# Supplementary material for: Nuclear translocation of FGFR1 and FGF2 in pancreatic stellate cells facilitates pancreatic cancer cell invasion
Source: EMBO Mol Med. 2014 Feb 6;6(4):467–81. doi: 10.1002/emmm.201302698 (PMC3992074; doi:10.1002/emmm.201302698)
Supplement: Supplementary file 14 [file emmm0006-0467-sd14.pdf]

Supporting Information Table 3

On Target plus SMARTPOOL siRNA FGFR1 L-003131	Target Sequence
J-003131-10	GCCACACUCUGCACCGCUA
J-003131-11	CCACAGAAUUGGAGGCUAG
J-003131-12	CAAAUGCCCUUCCAGUGGG
J-003131-13	GAAAUUGCAUGCAGUGCCG
**On Target plus SMARTPOOL siRNA FGF2 L-006695**	**Target Sequence**
J-006695-05	CUAAAUGUGUUACGGAUGA
J-006695-06	UCAAAGGAGUGUGUGCUAA
J-006695-07	GCUAAGAGCUGAUUUUAAU
J-006695-08	GAUGGAAGAUUACUGGCUU
**On Target plus SMARTPOOL siRNA FRS2**	**Target Sequence**
J-060152-05	GGUGGGAAGUGCUCGCUUA
J-060152-06	GAGAAGACCUGCACUAUUA
J-060152-07	UGAGAGAACUGCUGCUAUG
J-060152-08	GUACACCGACAGUCUUUAA
