# Supplementary material for: Nuclear translocation of FGFR1 and FGF2 in pancreatic stellate cells facilitates pancreatic cancer cell invasion
Source: EMBO Mol Med. 2014 Feb 6;6(4):467–81. doi: 10.1002/emmm.201302698 (PMC3992074; doi:10.1002/emmm.201302698)
Supplement: Supplementary file 15 [file emmm0006-0467-sd15.pdf]

Supporting Information Fig 1. Nuclear signal co-localisation with confocal microscopy.

A. Human pancreatic tissues were stained with antibodies to FGF2 (red) and cytokeratin (green) and counter-stained with DAPI (blue). Using confocal microscopy, intensity thresholds for FGF2 and DAPI pixels were standardised across all images and analysed using the cross-hair function (see Methods). White pixels (arrow) depict co-localised FGF2 and DAPI pixels (as demonstrated in third quadrant in the adjacent graph). Co-localisation is obvious in cytokeratin-negative cells, and absent in the cytokeratin-positive cancer cells.

B. There was no correlation between percentages of cancer cells with nuclear FGF2 and FGFR1 from 36 patients who had been scored for both FGFR1 and FGF2. Each data point represents one patient.
