# Supplementary material for: Nuclear translocation of FGFR1 and FGF2 in pancreatic stellate cells facilitates pancreatic cancer cell invasion
Source: EMBO Mol Med. 2014 Feb 6;6(4):467–81. doi: 10.1002/emmm.201302698 (PMC3992074; doi:10.1002/emmm.201302698)
Supplement: Supplementary file 16 [file emmm0006-0467-sd16.pdf]

Supporting Information Fig 2. FGFR1 and FGF2 expression in pancreatic cancer, normal ductal epithelial and stellate cell lines.

A-D. Coverslip-plated cells were stained with antibodies to FGFR1 (green) or FGF2 (red), nuclei were counter stained with DAPI (blue). Poorly- (A: AsPc-1, MIA PaCa-2, PANC-1, PaTu8898S, Hs766T, SUIT-2) and well-differentiated (B: Capan-1, Capan-2, CFPAC-1, 818.1, COLO-357, PaTu8898T) pancreatic cancer cell lines showed cytoplasmic or peri-nuclear FGF2 staining (arrow head). Most of the poorly-differentiated cells lines showed strong nuclear FGFR1, whereas in well-differentiated cell lines FGFR1 was mainly cytoplasmic (arrow). In contrast, FGFR1 and FGF2 were absent from the nuclei of control normal epithelial cell lines HPDE and DEC-hTERT (C). Stellate cells (PS1, D) showed strong nuclear FGF2 and FGFR1 (yellow arrow head).

E. Immunoblot analyses were performed with lysates from poorly- and well-differentiated, normal ductal epithelial (HPDE and DEC-hTERT), pancreatic stellate (PS1) cell lines. MCF-7 breast cancer cell line was used as a positive control. Tubulin was used as a loading control. Pancreatic cancer cell lines showed moderate expression of high molecular weight (HMW, 24 kDa) and low molecular weight (LMW, 18 kDa) forms of FGF2, however the majority of well-differentiated cell lines showed no expression of either isoform of FGF2. Weak expression of FGF2 was detected in control cell lines HPDE and DEC-hTERT. PS1 cells showed strong expression of both HMW (24 kDa) and LMW (18 kDa) forms of FGF2. Whilst FGFR1 expression was weak in normal epithelial cell lines, its expression appeared higher in many poorly-differentiated cell lines.

Scale Bar: 20 µm.
