# Supplementary material for: Nuclear translocation of FGFR1 and FGF2 in pancreatic stellate cells facilitates pancreatic cancer cell invasion
Source: EMBO Mol Med. 2014 Feb 6;6(4):467–81. doi: 10.1002/emmm.201302698 (PMC3992074; doi:10.1002/emmm.201302698)
Supplement: Supplementary file 17 [file emmm0006-0467-sd17.pdf]

Supporting Information Fig 3. FGFR1 and FGF2 expression in primary pancreatic stellate cells.

A. Pancreatic stellate cells (PSCs) isolated from resected human cancer specimens (Methods) were characterised by expression of αSMA, GFAP, desmin and vimentin markers (red).

B. Primary pancreatic stellate cells (PSC1, HPSC, FS1) as well as a pancreatic stellate cell line (RLT-PSC) demonstrated speckled nuclear localisation of FGFR1 (green arrow) as well as diffuse nuclear FGF2 (red, arrow).

Scale Bar: 20 μm.
