# Supplementary material for: Nuclear translocation of FGFR1 and FGF2 in pancreatic stellate cells facilitates pancreatic cancer cell invasion
Source: EMBO Mol Med. 2014 Feb 6;6(4):467–81. doi: 10.1002/emmm.201302698 (PMC3992074; doi:10.1002/emmm.201302698)
Supplement: Supplementary file 18 [file emmm0006-0467-sd18.pdf]

Supporting Information Fig 4. FGFR1 and FGF2 RNAi in stellate and cancer cell lines.

A. Cell lines (PS1, MIA PaCa-2 and COLO-357) were transfected with either FGF2 siRNA (FGF2) or scrambled non-targeting (Scr) siRNA for 72 hours. FGF2 expression levels were analysed by immunoblotting. Tubulin was used as a loading control. The ratio of FGF2 (HMW, 24kDa and LMW, 18kDa) to Tubulin was determined by densitometric analysis. FGF2 RNAi treatment achieved >90% knock-down of FGF2 protein levels in PS1, MIA-PaCa-2 and COLO-357 cells. PS1, *P=0.0148, **P= 0.0033. MIA PaCa-2, *P= 0.0171,**P=0.0010. COLO-357, *P=0.0319. Student's t-test. Data summary represented by mean ± SEM.

B. MIA PaCa-2 and COLO-357 cells were treated with FGF2 siRNA for 72 hours and stained with antibodies to FGF2 and FGFR1. Treatment with FGF2 siRNA resulted in significant reduction in FGF2 expression but no effect was seen on nuclear FGFR1 levels. MIA PaCa-2 **P= 0.0015. COLO-357 **P=0.0020. Student's t-test. Data summary represented by mean ± SEM.

C. PS1 and COLO-357 cells were transfected with FGFR1 siRNA (FGFR1) or scrambled non-targeting siRNA (Scr) for 72 hours and then lysed for immunoblotting to analyse expression levels of FGFR1. HSC70 was used as a loading control. The ratio of full length FGFR1 (160 kDa) to HSC70 was determined by densitometric analysis..*P=0.0478, **P=0.0041. Student's t-test. Data summary represented by mean ± SEM

Scale Bar: 20 µm

Images are representative of at least three independent experiments.
