# Supplementary material for: Nuclear translocation of FGFR1 and FGF2 in pancreatic stellate cells facilitates pancreatic cancer cell invasion
Source: EMBO Mol Med. 2014 Feb 6;6(4):467–81. doi: 10.1002/emmm.201302698 (PMC3992074; doi:10.1002/emmm.201302698)
Supplement: Supplementary file 19 [file emmm0006-0467-sd19.pdf]

Supporting Information Fig 5. Abolishing FGF2 in cancer cells had no effect on nuclear FGFR1

A. Sub-cellular fractionation and subsequent immunoblotting of lysates from COLO-357 cells showed efficient knock-down of cytoplasmic FGF2 with absence of nuclear FGF2 and with no effect on nuclear FGFR1. Total lysate was used as a positive control. Lamin A/C and tubulin were used as markers of fraction purity and loading control.
B. Treatment of PS1 cells with increasing concentration of PD173074 (2nm to 2µm) leads to a significant reduction in nuclear FGFR1. ***P=0.0007. Kruskal-Wallis test with Dunn's post-test comparison. Data summary represented by mean ± SEM
C. Quantification following increasing concentrations of PD173074 shows significant reduction of nuclear FGFR1 in PS1 cells after 100µM and 2µM PD173074 tretament compared to DMSO control.
D. COLO-357 cells were treated with PD173074 for 48 hours and stained with antibodies to GFR1. No effect was seen on nuclear FGFR1. Students t test. Data summary represented by mean ± SEM.

Scale Bar: 20 µm

Data summary represented by mean ± SEM. Images are representative of at least three independent experiments.
