# Supplementary material for: Nuclear translocation of FGFR1 and FGF2 in pancreatic stellate cells facilitates pancreatic cancer cell invasion
Source: EMBO Mol Med. 2014 Feb 6;6(4):467–81. doi: 10.1002/emmm.201302698 (PMC3992074; doi:10.1002/emmm.201302698)
Supplement: Supplementary file 20 [file emmm0006-0467-sd20.pdf]

Supporting Information Fig 6. FGF2-specific FGFR signalling mediated by FRS2 and MAPK/MEK/ERK pathway.

A. Treatment of PS1 cells with FGF2 led to an increase in pERK and pFRS2 levels within 15 minutes. Activation of ERK and FRS2 was completely abolished following pre-treatment of PS1 cells with PD174074 inhibitor for 1 hour prior to FGF2 stimulation.

B-D. PS1 cells were treated with FRS2 RNAi and stained with antibodies to FGFR1 and FGF2. Knock down of FRS2 leads to a significant reduction in both nuclear FGFR1 and FGF2 (quantified in C and D, respectively) C, *P=0.0112, **P=0.0012. Students t-test. Data summary represented by mean ± SEM.

E-F. Efficient knockdown of FRS2 after 72 hours was confirmed using Western blotting. Quantification of densitometry is shown in F.

E. G-H. PS1 cells were serum starved for 12 hours and subsequently stimulated with exogenous recombinant FGF2 (100µg/ml) for 2 hours. After 15 minutes of stimulation, there was a significant increase in both nuclear FGFR1 and FGF2 compared to serum starved cells. ***P<0.0001. Kruskal-Wallis test with Dunn's post-test comparison. Data summary represented by mean ± SEM

Scale Bar: 20 µm

Images are representative of at least three independent experiments
