# Supplementary material for: Nuclear translocation of FGFR1 and FGF2 in pancreatic stellate cells facilitates pancreatic cancer cell invasion
Source: EMBO Mol Med. 2014 Feb 6;6(4):467–81. doi: 10.1002/emmm.201302698 (PMC3992074; doi:10.1002/emmm.201302698)
Supplement: Supplementary file 21 [file emmm0006-0467-sd21.pdf]

Supporting information Fig 7. Nuclear FGFR1 and FGF2 correlate with Ki67 positivity in PSCs

A. Shows that all cells that were Ki67 positive (green) showed nuclear FGF2 (red).

B. Similarly, all cells that were Ki67 positive (green) showed nuclear FGFR1

C. Graphs show significant correlation between percentage of PS1 cells with nuclear FGF2 and Ki67 as well as percentage of PS1 cells with nuclear FGF2 and Ki67.

Scale Bar: 20 µm

Data summary represented by mean ± SEM. Images are representative of at least three independent experiments
