# Supplementary material for: Nuclear translocation of FGFR1 and FGF2 in pancreatic stellate cells facilitates pancreatic cancer cell invasion
Source: EMBO Mol Med. 2014 Feb 6;6(4):467–81. doi: 10.1002/emmm.201302698 (PMC3992074; doi:10.1002/emmm.201302698)
Supplement: Supplementary file 22 [file emmm0006-0467-sd22.pdf]

Supporting Information Fig 8. Cancer cell proliferation following FGF2 and FGFR1 knock-down.

A-C. FGF2 RNAi in COLO-357 cells had no effect on proliferative fraction (percentage Ki67 positive cells: green) or total cell number. Students t test. Data summary represented by mean ± SEM.

D-F. FGFR1 RNAi in COLO-357 cells had no effect on proliferative fraction (percentage Ki67 positive cells: green) or total cell number. Students t test. Data summary represented by mean ± SEM.

G-I. Chemical inhibition of FGFR1 signalling (PD173074, 2 μM) in COLO-357 cells had no effect on proliferative fraction (percentage Ki67 positive cells: green) or cell growth after 5 days treatment compared to vehicle (DMSO) treated cells. Students t test. Data summary represented by mean ± SEM.

J. FGFR1 knock-down had no effect on expression of either HMW (24 kDa) or LMW (18 kDa) isoforms of FGF2 compared to Scr RNAi at 72 hours post-transfection in COLO-357 cells. Students t test. Data summary represented by mean ± SEM.

K. FGFR chemical inhibition (PD173074, 2 μm, 48 hours), had no effect on Cyclin D1 expression in COLO-357 cells compared to vehicle control. Students t test. Data summary represented by mean ± SEM.

Scale Bar: 20 μm.

n.s. not significant. Images are representative of at least three independent experiments.
