# Supplementary material for: Nuclear translocation of FGFR1 and FGF2 in pancreatic stellate cells facilitates pancreatic cancer cell invasion
Source: EMBO Mol Med. 2014 Feb 6;6(4):467–81. doi: 10.1002/emmm.201302698 (PMC3992074; doi:10.1002/emmm.201302698)
Supplement: Supplementary file 23 [file emmm0006-0467-sd23.pdf]

Supporting Information Fig 9. Invasion of primary stellate and cancer cells in a mini-organotypic 3D model.

A. Schematic model of mini-organotypic culture model. Transwell inserts (0.4 μm) were coated with Matrigel and Collagen. Cancer cells (COLO-357) were admixed with primary PSCs in a 1:2 ratio and cultured on top of the ECM. Cultures were fed from below with either medium containing PD173074 (2 μM) or DMSO vehicle control. Cultures were harvested at day 7.

B. COLO–357 cells only (or primary PSCs and COLO-357 cells) were cultured in the presence or absence of PD173074 for 7 days. H&E staining of gel sections showed that COLO-357 cells alone formed a thin monolayer on top of the ECM and were not affected by treatment with PD173074. When primary PSCs and COLO-357 cells were cultured together there was a marked increase in cell number and invasion into the ECM, which was abrogated by treatment with PD173074.

C. Vimentin (red) and cytokeratin (green) staining showed that both cancer cells (cytokeratin positive) and primary PSCs (vimentin positive) invaded into the ECM) and this was blocked by PD173074 treatment.

D. FGFR1 (green) appears nuclear in invading stellate cells (vimentin positive) when treated with vehicle control compared to stellate cells that failed to invade following treatment with PD173074. These images are zoomed in to demonstrate the invading stellate cells in the ECM for the DMSO control and stellate cells next to cancer cells in the PD173074 treated mini-organotypics.

Scale bar 100 μm.
