# Supplementary material for: Nuclear translocation of FGFR1 and FGF2 in pancreatic stellate cells facilitates pancreatic cancer cell invasion
Source: EMBO Mol Med. 2014 Feb 6;6(4):467–81. doi: 10.1002/emmm.201302698 (PMC3992074; doi:10.1002/emmm.201302698)
Supplement: Supplementary file 25 [file emmm0006-0467-sd25.pdf]

Supporting Information Fig 10. Stellate cell proliferation upon conditioned media from cancer cells

A Shows an increase in nuclear FGFR1 (green) and FGF2 (red) following culture of PS1 cells with conditioned media (serum-free) from COLO-357 cancer cells and complete media (10% FBS) compared to conditioned media from Dec hTERT normal epithelial cells or PS1 cells cultured in serum-free media (control).

B Quantification of percentage of PS1 cells with nuclear FGF2 shows that there was a significant increase in nuclear FGF2 following culture of PS1 cells with conditioned media from COLO-357 cells or complete media (10% FBS) compared to conditioned media from Dec hTERT normal epithelial cells (normalised to PS1 cells cultured in serum-free media). ***P<0.0001. Kruskal-Wallis test with Dunn's post-test comparison. Data summary represented by median ± interquartile range

C Quantification of percentage of PS1 cells with nuclear FGFR1 shows that there was a significant increase in nuclear FGFR1 following culture of PS1 cells with conditioned media from COLO-357 cells or complete media (10% FBS) compared to conditioned media from Dec hTERT normal epithelial cells (normalised to PS1 cells cultured in serum-free media). ***P<0.0001. Kruskal-Wallis test with Dunn's post-test comparison. Data summary represented by median ± interquartile range

Scale bar 20 μm. Images are representative of at least three independent experiments.
