# Supplementary material for: Nuclear translocation of FGFR1 and FGF2 in pancreatic stellate cells facilitates pancreatic cancer cell invasion
Source: EMBO Mol Med. 2014 Feb 6;6(4):467–81. doi: 10.1002/emmm.201302698 (PMC3992074; doi:10.1002/emmm.201302698)
Supplement: Supplementary file 26 [file emmm0006-0467-sd26.pdf]

Supporting Information

Table of contents

Supporting Information Figure 1

Supporting Information Figure 2

Supporting Information Figure 3

Supporting Information Figure 4

Supporting Information Figure 5

Supporting Information Figure 6

Supporting Information Figure 7

Supporting Information Figure 8

Supporting Information Figure 9

Supporting Information Figure 10

Supporting Information Figure 1 Legend

Supporting Information Figure 2 Legend

Supporting Information Figure 3 Legend

Supporting Information Figure 4 Legend

Supporting Information Figure 5 Legend

Supporting Information Figure 6 Legend

Supporting Information Figure 7 Legend

Supporting Information Figure 8 Legend

Supporting Information Figure 9 Legend

Supporting Information Figure 10 Legend

Supporting Table 1

Supporting Table 2

Supporting Table 3
